# Immunoregenerative Microenvironment Modulation in Tendon–Ligament Regeneration: Evolution of Chitosan-Based Composite Biomaterials

**DOI:** 10.3390/ijms27198546

**Published:** 2026-09-24

**Authors:** Muhammad Wibowo Ariyanto, Zairin Noor, Dwikora Novembri Utomo, Oski Illiandri

**Affiliations:** 1 Doctoral Program, Faculty of Medicine, Lambung Mangkurat University, Banjarbaru 70714, Indonesia; wibowo.ariyanto@ulm.ac.id (M.W.A.); oilliandri@ulm.ac.id (O.I.); 2 Department of Orthopaedics and Traumatology, Faculty of Medicine, Lambung Mangkurat University, Banjarmasin 70233, Indonesia; 3 Department of Orthopaedics and Traumatology, Faculty of Medicine, Airlangga University, Surabaya 60286, Indonesia; dwikora-novembri-u@fk.unair.ac.id

**Keywords:** chitosan, immunomodulation, tendon–ligament regeneration, immune microenvironment, composite biomaterials, tissue engineering

## Abstract

Tendon and ligament injuries remain challenging to treat because healing may result in fibrosis, incomplete tendon–bone integration, impaired biomechanical properties, and reinjury. This review critically synthesizes preclinical evidence on chitosan-based composite biomaterials for tendon–ligament regeneration, focusing on their evolution from structural scaffolds toward multifunctional immunoregenerative platforms. Evidence across immune regulation, inflammatory signaling, angiogenesis, progenitor-cell activity, extracellular matrix remodeling, fibrocartilage formation, enthesis regeneration, tendon–bone healing, and biomechanical recovery was evaluated, while distinguishing direct chitosan-related evidence from contextual evidence derived from broader biomaterial systems. Chitosan-based platforms may support multiple regenerative domains through structural guidance, bioadhesion, localized delivery of bioactive components, and formulation-dependent immunomodulatory activity. However, heterogeneity in chitosan physicochemical properties, composite formulations, experimental models, and outcome measures limits direct comparison and prevents definitive attribution of multicomponent effects to chitosan alone. We propose the Immunoregenerative Cascade Theory and Immunoregenerative Tendon–Ligament Unit as complementary, hypothesis-generating frameworks for organizing temporal and spatial–functional interactions during regeneration. These frameworks are not validated causal or predictive models. Future research should prioritize standardized biomaterial characterization, longitudinal molecular–histological–biomechanical assessment, emerging multi-omics approaches, clinically relevant large-animal validation, and mechanism-guided biomaterial design to establish whether immunoregulatory effects translate into durable structural and functional tendon–ligament regeneration with long-term reproducible clinically meaningful recovery outcomes.

## 1. Introduction

### 1.1. Clinical Challenges in Tendon–Ligament Regeneration

Tendon and ligament injuries remain a major cause of pain, functional impairment, joint instability, reduced physical performance, and long-term disability [1,2,3,4,5]. Their clinical burden is particularly relevant in commonly affected structures such as the rotator cuff, Achilles tendon, anterior cruciate ligament (ACL), and posterior cruciate ligament (PCL), where incomplete healing may contribute to recurrent injury, persistent instability, impaired tendon–bone integration, and degenerative joint changes despite advances in reconstruction, fixation, and rehabilitation [2,3,4,5,6,7,8]. The limited regenerative capacity of tendon and ligament tissues is related to their relatively low cellularity and vascularity, slow metabolic turnover, and highly organized extracellular matrix architecture [1,2,3,5,9]. Following injury, healing frequently results in fibrotic tissue with disorganized collagen architecture, incomplete fibrocartilage formation, impaired interface reconstruction, and mechanical properties that remain inferior to native tissue [1,2,3,4,10,11,12,13].

Importantly, regenerative failure cannot be explained solely by structural disruption or inadequate mechanical reconstruction [1,9,11,12,13]. Tendon–ligament healing involves dynamic interactions among immune cells, stromal cells, vascular networks, cytokines, growth factors, extracellular matrix components, and biomechanical signals that collectively influence the quality and durability of tissue repair [1,9,11,13,14]. These observations have encouraged a broader therapeutic perspective in which structural reconstruction is complemented by strategies aimed at modulating the biological microenvironment rather than relying exclusively on mechanical restoration [3,9,13,14,15,16,17,18].

### 1.2. Immune Microenvironment as a Therapeutic Target

The immune microenvironment plays a major regulatory role in tendon–ligament healing because inflammatory responses influence early tissue debridement, cellular recruitment, vascular adaptation, extracellular matrix remodeling, and subsequent tissue maturation [1,9,11,12,13]. Acute inflammation is necessary for debris clearance and initiation of repair, whereas prolonged or dysregulated inflammatory activity may sustain cytokine imbalance, matrix degradation, fibrosis, oxidative stress, and incomplete tissue regeneration [1,9,10,14,16]. Macrophages are particularly relevant because their phenotypic plasticity allows them to participate in inflammatory resolution, angiogenesis, progenitor-cell recruitment, extracellular matrix remodeling, and interface maturation [10,11,12,13,14]. Successful healing therefore depends not on complete suppression of inflammation but on coordinated progression toward a pro-resolving regenerative microenvironment [11,12,13,14,16].

This transition is associated with changes in cytokine networks, intracellular signaling, oxidative-stress responses, angiogenic mediators, mechanotransduction, and extracellular matrix-derived signals that collectively shape downstream regenerative events [9,11,12,13,14]. Persistent activation of inflammatory pathways such as NF-κB and NLRP3 may contribute to maladaptive tissue remodeling, whereas timely resolution of inflammatory signaling may support constructive healing [4,10,16,19,20,21]. Accordingly, the immune microenvironment should be regarded as a modifiable therapeutic target within a broader regenerative system rather than as the sole determinant of tissue regeneration. Vascularization, progenitor-cell activity, extracellular matrix remodeling, mechanical loading, and tissue-specific structural cues interact bidirectionally with immune processes to influence regenerative outcomes. Immunoregenerative biomaterials should therefore be viewed as modulators of this interconnected system rather than as independent determinants of tissue regeneration.

### 1.3. Evolution of Chitosan-Based Biomaterials

Regenerative biomaterial design has progressively evolved from predominantly structural scaffolds toward bioactive and biologically responsive platforms as understanding of tendon–ligament healing has expanded beyond mechanical support alone [2,3,5,15,22]. Early biomaterials were primarily designed to restore mechanical continuity, provide temporary structural support, and facilitate cellular attachment and tissue organization [23,24,25,26]. Although these approaches established an important foundation for tissue engineering, their predominantly structural function provided limited control over the biological processes that determine matrix remodeling, vascular adaptation, cellular recruitment, and tissue integration [1,3,9,10,11,23,24,25,26]. This limitation stimulated the development of bioactive biomaterials incorporating extracellular matrix components, platelet concentrates, growth factors, hydrogels, bioactive coatings, nanoparticles, and controlled-release systems to provide localized biological cues in addition to structural support [27,28,29,30,31]. Within this transition, chitosan emerged as an adaptable biomaterial because of its biocompatibility, biodegradability, bioadhesion, chemical versatility, tunable physicochemical properties, and compatibility with diverse scaffold and delivery configurations [22,32,33,34,35,36].

The subsequent development of immunoregenerative biomaterials reflects increasing recognition that biomaterial–host interactions and the immune microenvironment influence multiple stages of tissue regeneration [1,9,11,12,13,14,15]. Rather than serving solely as passive structural matrices or vehicles for bioactive factors, newer platforms are increasingly designed to modulate inflammatory resolution while simultaneously supporting cellular recruitment, angiogenesis, extracellular matrix remodeling, and tissue maturation [9,11,12,13,14,15,16]. Chitosan has also been investigated for its ability to influence macrophage behavior toward a pro-resolving phenotype, providing a biological basis for its incorporation into immunoregenerative biomaterial strategies [37]. Chitosan-containing systems have consequently been incorporated with platelet-derived products, extracellular matrix components, stem or progenitor cells, nanoparticles, growth factors, hydrogels, and controlled-delivery strategies to integrate structural and biological functions within a single regenerative platform [27,29,38,39,40]. These developments are consistent with the broader evolution of fibrous and multifunctional biomaterial systems for tendon–ligament repair and regeneration [41]. This evolution positions chitosan-based composites within the broader transition from scaffold-centered tissue engineering toward multifunctional immunoregenerative strategies, while recognizing that biological effects in multicomponent systems represent the combined contribution of chitosan, incorporated bioactive components, material processing, and host responses rather than the action of chitosan alone [3,9,17,18,27,29].

### 1.4. Objective and Scope of This Review

Current evidence on chitosan-based biomaterials for tendon–ligament regeneration remains heterogeneous with respect to material formulations, experimental models, biological targets, and outcome measures. This review aims to critically synthesize the available preclinical evidence on chitosan-based composite biomaterials for tendon–ligament regeneration, with particular emphasis on the relationship between biomaterial design, immune regulation, and integrated regenerative outcomes. The review encompasses evidence addressing immune-cell responses and inflammatory regulation, angiogenesis and progenitor-cell activity, extracellular matrix remodeling, fibrocartilage and enthesis formation, tendon–bone integration, and biomechanical restoration. The objective is not to establish a single dominant mechanism of regeneration but to evaluate how these interconnected biological and functional domains are represented across the available evidence.

A central scope of this review is the explicit distinction between direct chitosan-related evidence and contextual evidence from broader biomaterial and regenerative-medicine research. Findings obtained from chitosan or chitosan-containing platforms are considered direct evidence, whereas studies involving non-chitosan biomaterials or broader regenerative systems are used only as contextual evidence to support mechanistic interpretation or comparison where chitosan-specific evidence is limited. This distinction is maintained throughout the evidence synthesis to avoid attributing effects of multicomponent systems or non-chitosan platforms exclusively to chitosan. Accordingly, the review evaluates the consistency, mechanistic relevance, and translational implications of the available evidence while recognizing the limitations imposed by heterogeneity across biomaterial formulations, experimental models, and outcome measures.

### 1.5. Literature Search and Evidence Synthesis

This review was designed as a critical narrative review rather than a systematic review or meta-analysis. Accordingly, the literature search was intended to provide a focused and representative synthesis of evidence relevant to chitosan-based tendon–ligament regeneration rather than an exhaustive systematic identification of all eligible studies. Relevant literature was identifiedfrom Scopus, PubMed, and Google Scholar using combinations of terms related to chitosan, tendon, ligament, tissue engineering, immunomodulation, macrophage, inflammation, angiogenesis, extracellular matrix, and tendon–bone healing. The literature search covered publications from January 2016 to May 2026, with emphasis on preclinical studies and mechanistically relevant evidence. Priority was given to preclinical studies directly evaluating chitosan or chitosan-containing composite systems in tendon–ligament regeneration, while mechanistically relevant studies from broader biomaterial and regenerative-medicine research were considered when direct chitosan-specific evidence was limited.

Evidence was synthesized qualitatively according to its directness to chitosan-based systems, consistency across studies, mechanistic support, integration of biological and functional outcomes, and translational relevance. Direct evidence from chitosan-containing systems was distinguished from contextual evidence derived from non-chitosan biomaterials or broader regenerative models, which was used only to support mechanistic interpretation and not as evidence of chitosan-specific effects. The resulting evidence hierarchy therefore represents a structured qualitative synthesis rather than a formal certainty-of-evidence assessment, quantitative ranking, or meta-analytic comparison.

## 2. Evolution of Chitosan-Based Composite Biomaterials

The development of biomaterials relevant to tendon–ligament regeneration has progressively expanded from predominantly structural scaffolds toward bioactive and increasingly immunoregulatory platforms. This conceptual evolution reflects growing recognition that successful tissue repair depends not only on mechanical support but also on interactions among biomaterial properties, host immune responses, vascular remodeling, cellular activity, and extracellular matrix organization [2,3,9,11,14,15]. Within this evolving landscape, chitosan has been incorporated into diverse composite systems that combine structural, delivery, and biological functions, although the regenerative effects of multicomponent platforms should not be attributed exclusively to chitosan [22,27,29,32]. The overall conceptual evolution of these biomaterial strategies is illustrated in Figure 1.

### 2.1. Structural Biomaterials

Early biomaterials developed for tendon–ligament repair were designed predominantly to restore mechanical continuity, provide temporary structural support, and facilitate cellular infiltration and tissue organization at the injury site [2,3,5,23,24,25,26,41]. Natural grafts, synthetic ligaments, electrospun fibers, and polymer-based scaffolds provided physical matrices for cellular attachment, collagen deposition, and early tissue stabilization, representing important advances in structural tissue engineering [23,24,25,26,41]. However, predominantly structural approaches do not necessarily reproduce the native hierarchical organization of tendon and ligament tissues, and healing may remain characterized by disorganized collagen architecture, fibrotic tissue formation, incomplete fibrocartilage development, and suboptimal tendon–bone integration [1,3,4,10,11,12,23,24,25,26]. These limitations indicate that mechanical support alone may be insufficient to reproduce the biological and functional complexity of native tendon–ligament regeneration [2,3,5,9,11].

Recognition of these limitations contributed to increasing interest in biomaterials capable of providing biological cues in addition to structural support [3,9,14,17,22]. Rather than implying that early scaffolds intrinsically failed because they lacked immune-regulatory activity, available evidence supports a broader interpretation in which regenerative outcome depends on interactions among scaffold architecture, material properties, host inflammatory responses, vascularization, extracellular matrix remodeling, and mechanical loading [1,9,11,12,13,14,15]. This evolving understanding provided the rationale for developing bioactive biomaterials designed to combine structural functions with localized biological signaling during tissue repair [3,14,15,17,22].

### 2.2. Bioactive Biomaterials

Recognition of the biological complexity of tendon–ligament healing stimulated development of bioactive biomaterials intended to complement structural support with biological signals that promote cellular recruitment, matrix synthesis, angiogenesis, and tissue integration [2,3,5,9,15]. These platforms incorporated extracellular matrix derivatives, platelet concentrates, growth factors, hydrogels, bioactive coatings, ceramics, and natural polymers, thereby expanding biomaterial function beyond predominantly mechanical support [27,28,29,30,31]. Chitosan became increasingly investigated within this context because of its biocompatibility, biodegradability, bioadhesion, chemical modifiability, and capacity to serve as a scaffold and delivery matrix [22,32,33,34,36]. Chitosan-containing systems have subsequently been combined with platelet-derived products, hydrogels, cells, nanoparticles, and controlled-delivery strategies to support localized bioactive retention, cellular activity, and extracellular matrix organization [27,29,38,39,40].

These developments support the broader principle that regenerative performance reflects interactions between biomaterial properties and the biological microenvironment rather than material composition alone [3,9,11,12,13,14]. However, biological effects observed in multicomponent chitosan systems reflect the combined contributions of the scaffold, incorporated cells or bioactive agents, material processing, and host response and therefore should not automatically be attributed to chitosan itself [22,27,29,32,38,39,40]. Increasing recognition of the role of inflammation and immune–material interactions subsequently encouraged development of biomaterial strategies designed to more specifically investigate and modulate host immune responses during tissue regeneration [9,11,12,13,14,15].

### 2.3. Immunoregenerative Biomaterials

Advances in regenerative immunology have increased recognition that biomaterial–host immune interactions can influence multiple stages of tendon–ligament healing, including inflammatory resolution, vascular remodeling, extracellular matrix organization, and tissue maturation [1,9,11,12,13]. Accordingly, immunoregenerative biomaterials are being developed to combine structural or delivery functions with modulation of selected components of the host immune microenvironment [9,11,13,14,15,16]. Direct chitosan-related evidence indicates that chitosan’s physicochemical characteristics and formulations can influence macrophage responses and inflammatory signaling, including pathways involving NF-κB, NLRP3, and oxidative-stress regulation [19,20,21,33,37]. Chitosan-based bioadhesive systems have also been investigated for tendon-to-bone repair, demonstrating the integration of chitosan-mediated bioadhesion, mechanical competence, biocompatibility, and pro-tenogenic activity [42]. Chitosan-containing systems with additional immunomodulatory functions have further been investigated for suppression of NLRP3 inflammasome activity and promotion of tendon healing [43]. However, downstream effects involving angiogenesis, cellular recruitment, matrix remodeling, and functional tissue restoration often derive from chitosan-containing multicomponent systems or broader regenerative biomaterial studies and should therefore be interpreted according to their evidentiary directness [17,39,40,44,45].

Multifunctional regenerative platforms illustrate how immune modulation may be integrated with controlled delivery, vascular regulation, cellular support, extracellular matrix remodeling, and interface regeneration [14,17,18,43,46]. Importantly, evidence from non-chitosan immunoregulatory systems provides mechanistic and contextual support for these broader design principles but does not establish chitosan-specific effects, while outcomes from chitosan-containing composites may reflect contributions from multiple incorporated components [14,17,18,43,46]. Collectively, these findings support an emerging systems-oriented approach in which biomaterial performance is evaluated through interactions among material properties, immune cells, stromal cells, vascular networks, extracellular matrix components, and biomechanical conditions rather than through isolated material or molecular effects [3,9,12,13,14].

### 2.4. Why Chitosan?

Chitosan is of particular interest for tendon–ligament tissue engineering because it combines biocompatibility, biodegradability, bioadhesion, chemical modifiability, and compatibility with diverse scaffold and delivery configurations [22,32,33,34,36]. Importantly, its immunomodulatory activity is influenced by physicochemical characteristics rather than being an invariant property of chitosan itself. Molecular weight, degree of deacetylation, surface charge, particle size and morphology, solubility, and degradation behavior can modify the availability of protonated amino groups, material–cell interactions, cellular uptake, and the duration and magnitude of local exposure to chitosan-derived signals [19,20,21,22,32,33,37,43,47]. These structure–property characteristics may influence interactions between chitosan-based materials and immune cells and innate immune-sensing pathways, thereby modulating downstream signaling involving TLR4/MyD88–NF-κB, NLRP3 inflammasome activation, and oxidative-stress/Nrf2 responses. Consequently, changes in NF-κB- and NLRP3-associated inflammatory signaling and Nrf2-mediated antioxidant responses may alter macrophage activation states and the production of cytokines and inflammatory mediators, including TNF-α, IL-1β, and other context-dependent signaling molecules [19,20,21,33,37,43]. These effects should, however, be interpreted as formulation- and context-dependent rather than as evidence that chitosan uniformly acts as a direct agonist or antagonist of a single immune receptor. Direct chitosan-related evidence indicates that specific formulations can modulate macrophage behavior and inflammatory signaling, while multicomponent systems may additionally influence immune responses through incorporated platelet-derived products, cells, growth factors, nanoparticles, hydrogels, or other bioactive components [19,20,21,22,27,29,38,40,43,47,48]. Thus, the immunoregulatory potential of chitosan is best understood as a structure–property–immune response relationship, in which material characteristics influence the cellular and molecular context through which immunomodulatory effects emerge.

Chitosan should therefore be considered within the broader landscape of regenerative biomaterials rather than as intrinsically superior to alternative platforms. Collagen and decellularized extracellular matrix provide biologically instructive matrices that resemble native extracellular environments, whereas gelatin methacryloyl (GelMA) offers tunable hydrogel properties and compatibility with cell-based biofabrication. Synthetic polymers such as poly (lactic-co-glycolic acid) (PLGA) and polycaprolactone (PCL) provide controllable mechanical and degradation characteristics, while hyaluronic acid (HA) and silk fibroin offer complementary advantages in matrix interaction, biocompatibility, and structural engineering. Within this landscape, the rationale for focusing on chitosan lies in the combination of chemical modifiability, bioadhesion, biodegradability, delivery capacity, and formulation-dependent immunomodulatory activity, rather than demonstrated universal superiority over alternative biomaterials [22,32,33,34,36]. Moreover, its physicochemical properties can be deliberately modified to influence degradation, local retention, cellular interaction, and biological responsiveness, providing a degree of design flexibility relevant to immunoregenerative applications [22,32,47].

Recent studies have further expanded chitosan-based platforms through multifunctional formulations incorporating platelet-rich plasma, thrombin, tendon-derived stem cells, and other bioactive components, with reported effects on tendon–bone healing, collagen organization, inflammatory modulation, and biomechanical recovery [49,50,51,52,53,54,55,56]. These studies also illustrate the increasing complexity of chitosan-based systems, in which the biological effects of chitosan may interact with those of incorporated cells, platelet-derived factors, growth factors, or other functional components. Accordingly, interpretation of these findings requires consideration of the experimental model, biomaterial architecture, formulation characteristics, and the extent to which chitosan-specific effects can be distinguished from those of other components [49,50,51,52,53,54,55,56]. These developments also highlight the importance of experimental model selection, scaffold architecture, biomaterial characterization, and standardized reporting when interpreting preclinical regenerative outcomes [57,58,59,60,61]. Taken together, these considerations provide the rationale for the evidence-based comparison of direct chitosan studies and contextual alternative biomaterial platforms summarized in Table 1.

## 3. Mechanisms of Immune Microenvironment Modulation

### 3.1. Immune-Cell Populations and Macrophage Polarization

Tendon–ligament healing is regulated by a dynamic and heterogeneous immune-cell environment rather than by macrophages alone. Following tissue injury, damage-associated molecular signals promote recruitment and activation of innate immune cells, including neutrophils, monocytes/macrophages, and mast cells, followed by participation of adaptive immune populations such as T lymphocytes and, to a lesser extent, B lymphocytes. Neutrophils contribute to the early inflammatory response, debris clearance, and antimicrobial defense, whereas recruited monocytes differentiate into macrophages that participate in inflammatory regulation, phagocytosis, tissue remodeling, and resolution. Mast cells may contribute to vascular and inflammatory signaling, while lymphocyte populations can modulate the inflammatory milieu and interactions between immune and resident stromal cells. However, the relative contribution of lymphocytes, mast cells, and other immune-cell populations to tendon–ligament regeneration remains less well characterized than that of macrophages [1,9,11,12,13]. Thus, immune regulation should be understood as a coordinated and temporally evolving network in which different immune-cell populations contribute distinct, overlapping, and context-dependent functions rather than as a macrophage-exclusive process.

Among these populations, macrophages represent one of the best-characterized regulators of tendon–ligament healing because of their functional plasticity and their capacity to coordinate inflammation, matrix remodeling, angiogenesis, and tissue repair [1,10,11,12,13]. The conventional M1/M2 framework provides a useful descriptive model but does not fully represent the phenotypic continuum and temporal heterogeneity of macrophages in vivo. During the early inflammatory phase, pro-inflammatory macrophage states are commonly associated with markers such as CD86, CD80, and inducible nitric oxide synthase (iNOS/NOS2), together with mediators including TNF-α and IL-1β, which contribute to inflammatory signaling, phagocytosis, and initial tissue debridement. As healing progresses, macrophages may acquire pro-resolving or pro-regenerative characteristics, commonly represented by CD206 (MRC1) and Arg1, together with IL-10 and TGF-β, which are associated with inflammatory resolution, extracellular matrix deposition, angiogenic support, and tissue remodeling [10,11,12,13]. CD68 is also widely used as a pan-macrophage marker, whereas CD86/CD80 and CD206 are commonly used as indicators of pro-inflammatory and pro-regenerative states, respectively. Nevertheless, these markers should be interpreted as phenotypic indicators rather than definitive identifiers of discrete macrophage populations, because macrophage states exhibit substantial plasticity and overlap in vivo.

Importantly, macrophage polarization should not be interpreted as a simple binary transition from M1 to M2. Pro-inflammatory macrophage activity is necessary during the early phase of repair for debris clearance and initiation of the regenerative response, whereas timely resolution and progression toward pro-resolving states may facilitate matrix organization, angiogenesis, fibrocartilage formation, and functional recovery [1,10,11,12,13]. Conversely, persistent inflammatory activation may sustain cytokine production, oxidative stress, matrix degradation, and fibrotic remodeling, while dysregulated or prolonged reparative macrophage activity may also contribute to maladaptive remodeling, depending on the biological context. This temporal plasticity is particularly relevant to immunoregenerative biomaterials because the therapeutic objective is not indiscriminate suppression of macrophages or permanent induction of a single phenotype but modulation of the timing, magnitude, and functional state of macrophage responses. Direct chitosan-specific and chitosan-containing composite evidence indicates that chitosan formulations can influence macrophage behavior and inflammatory signaling through innate immune pathways and the local regenerative microenvironment [19,20,21,33,37]. Evidence from broader immunoregulatory biomaterial systems further supports the principle that therapeutic benefit may arise from promoting timely immune resolution while preserving early inflammatory functions required for repair; however, such contextual evidence should not be interpreted as demonstrating chitosan-specific effects [11,12,14,16,43]. Accordingly, the immunoregenerative potential of chitosan-based platforms is more appropriately interpreted as modulation of a dynamic immune network that coordinates macrophage responses with matrix remodeling and functional tissue repair rather than as a simple anti-inflammatory or M1-to-M2 switching effect.

### 3.2. Cytokine Regulation

The biological consequences of macrophage polarization are mediated through dynamic cytokine and chemokine networks that coordinate communication among immune cells, fibroblasts, vascular cells, progenitor cells, and extracellular matrix components throughout tendon–ligament healing [1,9,11,12,13]. Relevant chemokines, including C–C motif chemokine ligand 2 (CCL2) and stromal cell-derived factor-1 (SDF-1/CXCL12), contribute to the recruitment and regulation of macrophages and progenitor cells during tendon–ligament healing [12]. During early inflammation, TNF-α, IL-1β, and IL-6 contribute to immune-cell recruitment, debris clearance, and matrix turnover, whereas prolonged inflammatory signaling may disrupt collagen organization, tenogenic differentiation, tendon–bone integration, and functional repair [1,10,11,14,16]. Conversely, pro-resolving cytokines and regenerative growth factors, including IL-10, TGF-β, VEGF, and FGF-related signaling, contribute to inflammatory resolution, angiogenesis, extracellular matrix synthesis, fibrocartilage formation, and interface maturation [17,28,30,31,49]. These observations support a model in which successful regeneration depends on spatiotemporal regulation of cytokine networks rather than indiscriminate suppression of inflammatory mediators [1,9,11,12,13].

Within this regulatory context, chitosan-based composites can provide localized platforms for immunomodulation and sustained delivery of platelet-derived or incorporated bioactive factors [27,29,38,47,50,51,52]. Chitosan-containing matrices and hydrogel systems may prolong local retention and release of regenerative mediators, whereas evidence from broader non-chitosan delivery systems provides contextual support for the general principle that sustained local signaling can coordinate angiogenesis, immune regulation, and extracellular matrix remodeling [17,18,30,48]. The available evidence therefore supports interpreting chitosan-based systems as modulators of the local cytokine and growth-factor microenvironment rather than attributing every downstream cytokine-mediated effect directly to chitosan itself [27,29,38,47,50,51,52]. This distinction separates direct evidence for chitosan-containing delivery platforms from broader mechanistic evidence supporting cytokine-network regulation in tendon–ligament regeneration [3,9,11,14].

### 3.3. Signaling Pathways

Immune microenvironment modulation during tendon–ligament repair involves interconnected signaling pathways regulating inflammatory resolution, oxidative stress, macrophage behavior, cell survival, angiogenesis, and extracellular matrix remodeling [9,11,12,13,16]. Among these networks, NF-κB and the NLRP3 inflammasome represent major inflammatory hubs whose sustained activation can amplify pro-inflammatory cytokine production, oxidative stress, matrix degradation, and persistent immune activation [16,19,20,21,43]. Conversely, regenerative pathways involving PI3K/Akt, Akt/GSK-3β, Nrf2, and growth-factor-dependent signaling contribute to cell survival, antioxidant defense, angiogenesis, tenogenic differentiation, extracellular matrix synthesis, and tissue maturation [17,30,40,44,45]. These pathways should therefore be interpreted as interacting components of a broader regulatory network rather than as independent molecular determinants of regenerative outcome [9,11,12,13,16].

Direct chitosan-related evidence supports the modulation of selected inflammatory and regenerative signaling pathways, including NF-κB/NLRP3-associated inflammatory responses and Nrf2-related antioxidant mechanisms, while chitosan-containing cell and hydrogel platforms have also been associated with Akt/GSK-3β-related regenerative signaling [19,20,21,40,44,45]. However, the strength and directness of evidence differ among individual pathways, and findings derived from multicomponent chitosan platforms should not be attributed exclusively to the chitosan component when other bioactive constituents may contribute to the observed response [40,44,45]. Accordingly, current evidence supports the broader interpretation that chitosan-based biomaterials can participate in coordinated modulation of inflammatory and regenerative signaling networks rather than suggesting that chitosan independently controls every pathway involved in tendon–ligament regeneration [14,19,20,21,40,44].

### 3.4. Angiogenic and Stem-Cell Recruitment

Angiogenesis and progenitor-cell recruitment represent interconnected downstream components of regenerative tissue repair because vascular networks restore oxygen and nutrient delivery while supporting immune-cell trafficking, progenitor-cell survival, extracellular matrix synthesis, and tissue maturation [3,5,11,17,28]. Proresolving macrophages and regenerative cytokine networks can contribute to this process through VEGF, TGF-β, and matrix-remodeling mediators that facilitate endothelial activation and the formation of a permissive regenerative niche [11,12,13,14,16]. Mesenchymal stem cells, tendon-derived stem/progenitor cells, and endogenous progenitor populations can subsequently contribute to collagen synthesis, tenogenic differentiation, fibrocartilage formation, and tendon–bone healing when maintained within an appropriately regulated microenvironment [2,3,39,40,53]. Thus, vascularization and progenitor-cell activity are more appropriately viewed as mutually reinforcing components of immune-guided regeneration than as independent therapeutic mechanisms [3,11,14,39].

Direct evidence from chitosan-containing hydrogels and cell-delivery systems indicates that these platforms can provide structural support, cellular retention, and localized bioactive environments conducive to progenitor-cell-mediated repair [38,39,40,53]. Chitosan-containing angiogenic composites further support the feasibility of integrating chitosan with growth-factor delivery and vascular-regenerative strategies, whereas findings from non-chitosan VEGF-, bFGF-, and other bioactive biomaterial systems should be regarded as contextual evidence for the broader biological value of sustained angiogenic signaling rather than as direct evidence of an intrinsic pro-angiogenic effect of chitosan [17,28,30,49,54]. Accordingly, the regenerative relevance of chitosan-based platforms lies in their capacity to serve as adaptable components of multifunctional systems that coordinate vascular support, progenitor-cell activity, immune regulation, and matrix reconstruction [38,39,40,53].

### 3.5. Immune–ECM Crosstalk

The extracellular matrix (ECM) is not merely the structural endpoint of tendon–ligament healing but an active component of the regenerative microenvironment that influences immune-cell behavior, mechanotransduction, cellular migration, and tissue remodeling [1,2,3,9,10]. Matrix stiffness, collagen architecture, degradation products, and biomechanical loading can modulate macrophage polarization, cytokine production, and fibroblast activity, while immune cells reciprocally regulate collagen turnover, MMP/TIMP balance, matrix organization, and scar formation [9,10,11,14,16]. Successful regeneration therefore depends on bidirectional immune–ECM communication leading to organized collagen type I deposition, fiber alignment, fibrocartilage development, tendon–bone interface maturation, and progressive biomechanical recovery [10,17,29,42,51]. This reciprocal relationship provides a mechanistic bridge between early immune regulation and subsequent structural and functional tissue maturation [9,10,11,14].

Direct evidence from chitosan-based scaffolds, bioadhesives, platelet composites, hydrogels, and electrospun constructs indicates that chitosan-containing platforms can provide structural guidance while simultaneously delivering localized biological cues that influence matrix organization and regenerative signaling [24,25,27,29,38]. More complex chitosan-containing and immunoregulatory composite systems further illustrate how structural support can be integrated with immune modulation, vascular remodeling, and functional tissue repair, although biological effects observed in multicomponent platforms cannot be attributed exclusively to chitosan [14,17,18,29,51]. Collectively, these findings support an integrative interpretation in which chitosan functions as one component of an immune–ECM regulatory platform capable of linking structural guidance with biological modulation, rather than as an independently acting determinant of all downstream regenerative processes [24,27,29,38]. These interconnected immunological, vascular, extracellular matrix, and biomechanical processes are integrated into the proposed Immunoregenerative Cascade Theory illustrated in Figure 2.

### 3.6. Immunoregenerative Cascade Theory

The Immunoregenerative Cascade Theory provides a temporal framework linking sequential regenerative events during tendon–ligament healing, beginning with early inflammation and its subsequent resolution, followed by coordinated vascular and cellular responses, extracellular matrix (ECM) remodeling, interface maturation, and ultimately biomechanical restoration [3,9,10,11,12,13,14,17]. Within this framework, the transition from inflammation to resolution establishes the biological conditions for vascular adaptation and cellular recruitment, which support progressive ECM organization and maturation, followed by reconstruction of the tendon–bone interface and restoration of mechanical competence. These stages are interconnected rather than strictly linear, with reciprocal interactions and feedback between immune, vascular, cellular, matrix, and mechanical processes throughout healing [1,9,11,13,15]. The Cascade Theory is therefore presented as the authors’ integrative, hypothesis-generating framework for organizing these temporal relationships rather than as a quantitatively validated causal or predictive model, with the corresponding design principles summarized in Table 2.

### 3.7. The Immunoregenerative Tendon–Ligament Unit (ITLU)

The Immunoregenerative Tendon–Ligament Unit (ITLU) is proposed as a spatial and functional framework describing the reciprocal interactions among immune, vascular, cellular, extracellular matrix (ECM), and biomechanical domains during tendon–ligament regeneration [1,3,9,11,13]. Rather than considering these components as independent determinants, the ITLU conceptualizes the healing site as an interconnected microenvironment in which immune-cell activity influences vascular adaptation and cellular behavior, vascular signals regulate cellular recruitment and tissue remodeling, and progenitor and stromal cells interact with the evolving ECM to influence tissue organization [9,12,13,14,17]. Conversely, changes in ECM architecture and mechanical loading can provide feedback signals that modify cellular and immune responses, thereby maintaining continuous communication among the major regenerative domains [1,3,10,11,12,13,14].

Within this framework, immune–vascular–cellular–ECM–mechanical interactions are considered spatially and functionally integrated throughout tissue repair rather than isolated biological events [3,9,12,13,14,15]. The ITLU therefore extends the interpretation of tendon–ligament regeneration beyond individual molecular pathways by emphasizing how local cellular interactions, vascular support, matrix organization, and mechanical cues collectively shape tissue adaptation and functional restoration. The ITLU is presented as the authors’ conceptual, hypothesis-generating framework and should not be interpreted as a validated predictive or causal model. The spatial and functional relationships represented by the ITLU are illustrated in Figure 3, whereas the corresponding evidence landscape across regenerative domains is summarized in Figure 4.

## 4. Biological Outcomes of Immune Modulation

### 4.1. ECM Remodeling

Extracellular matrix (ECM) remodeling represents a central structural outcome of tendon–ligament regeneration, with particular importance for collagen organization and overall ECM quality [1,3,9,10,11]. Successful healing requires a transition from a provisional, inflammation-associated matrix toward a more organized collagen-rich network with appropriate fibrillar architecture, composition, and maturation capable of supporting tissue integrity and physiological load transfer [2,3,10,29,51]. Persistent inflammatory activity may disrupt this process by promoting matrix degradation, excessive collagen type III deposition, disorganized collagen architecture, and fibrotic remodeling, thereby compromising the structural quality of the regenerated tissue [1,10,11,14,16]. Direct chitosan-related and chitosan-containing composite evidence indicates that chitosan-based platforms may support ECM organization through structural guidance, modulation of the local regenerative environment, and localized delivery of bioactive mediators [22,24,25,27,29,33,37]. Chitosan-containing platelet composites, hydrogels, and cell-based platforms have been associated with improved collagen organization and matrix maturation, although outcomes from multicomponent systems should not be attributed exclusively to chitosan [27,29,39,40,50]. Accordingly, the relevant regenerative endpoint is not simply increased collagen deposition but improved collagen architecture and ECM quality, which provide the structural basis for subsequent interface maturation and functional tissue restoration [10,14,17,29,55].

### 4.2. Fibrocartilage Formation

Fibrocartilage formation is a critical component of tendon–ligament regeneration because the native tendon–bone interface contains a specialized transition from tendon or ligament through unmineralized fibrocartilage and mineralized fibrocartilage to bone [2,3,11,17,28]. Proteoglycans and other matrix components contribute to the composition and functional properties of this transition, particularly within fibrocartilaginous regions subjected to complex tensile and compressive loading [2,3,11,17]. Following injury or reconstruction, inadequate fibrocartilage formation may result in replacement by mechanically inferior fibrous tissue and failure to reproduce the specialized matrix characteristics of the native interface [1,4,10,11,23]. Thus, assessment of fibrocartilage regeneration should consider not only the presence of fibrocartilaginous tissue but also proteoglycan-rich matrix formation and the development of both unmineralized and mineralized fibrocartilage regions.

Chitosan-containing platelet composites, hydrogels, and cell-based platforms may provide structural and biological environments that support fibrocartilage formation through localized mediator delivery, cellular retention, matrix organization, and lineage-specific differentiation [27,29,38,39,40,50,51,52,53]. Evidence from broader regenerative biomaterial systems further indicates that coordinated tenogenic, chondrogenic, and osteogenic signaling may contribute to the reconstruction of the fibrocartilaginous transition, although findings from non-chitosan systems should be interpreted as contextual rather than chitosan-specific evidence [17,30,31,49]. Interpretation of fibrocartilage-related outcomes should also consider differences in preclinical tendon–ligament models and experimental design, which may influence outcome comparability and translational interpretation [57]. Within chitosan-based platforms, fibrocartilage formation should therefore be interpreted according to the extent to which proteoglycan-associated matrix and unmineralized/mineralized fibrocartilage characteristics are restored rather than inferred solely from generalized histological improvement. This distinction is important because fibrocartilage maturation represents an intermediate structural step toward organized enthesis formation and subsequent tendon–bone integration [10,13,18,29,55].

### 4.3. Enthesis Regeneration

Enthesis regeneration represents a higher-order structural outcome requiring restoration of the zonal architecture of the tendon–bone interface, including the spatial transition from tendon or ligament through unmineralized fibrocartilage and mineralized fibrocartilage toward bone [2,3,4,11,17]. Successful reconstruction therefore requires appropriate spatial organization of these distinct tissue zones, together with corresponding cellular and extracellular matrix distributions that permit progressive changes in composition, mineralization, and mechanical properties across the interface [2,3,10,17,23]. Disruption of this spatial organization may produce a poorly graded fibrous interface and compromise mechanical continuity between soft tissue and bone [4,6,7,11,13]. Enthesis regeneration should consequently be evaluated not simply by the presence of fibrocartilage but by the organization, continuity, and spatial arrangement of the successive tissue zones that characterize a functionally competent enthesis.

Chitosan-based bioadhesives, platelet-containing composites, hydrogels, and other chitosan-containing platforms may support interface reconstruction through structural stabilization and localized delivery of bioactive mediators [27,29,42,50,51]. Evidence from chitosan-containing multifunctional systems and broader regenerative biomaterial platforms suggests that coordinated matrix guidance, angiogenic signaling, and osteochondral differentiation may contribute to restoration of features relevant to graded interface reconstruction; however, effects observed in multicomponent systems should not be attributed solely to chitosan [17,28,30,49,54]. The relevance of these platforms should therefore be considered in terms of their capacity to support spatially organized and progressively differentiated tissue architecture across the enthesis rather than isolated improvement in individual cellular or molecular endpoints [9,11,13,14,17]. Restoration of this zonal organization provides an important structural basis for effective biological integration and mechanical continuity between tendon–ligament tissue and bone [10,17,18,29,55].

### 4.4. Tendon–Bone Healing

Tendon–bone healing represents the functional integration of the regenerated soft-tissue and fibrocartilaginous interface with the receiving bone, making bone quality an important determinant of successful biological and structural integration [2,3,4,11,13]. The receiving bone should be regarded as an active regenerative substrate that provides mechanical anchorage, mineralized matrix, vascular support, and osteogenic capacity required for maturation of the tendon/ligament–fibrocartilage–bone interface [2,3,4,11,17]. Accordingly, successful tendon–bone healing requires adequate bone quality, bone remodeling, and osteogenic competence to support stable attachment and progressive mineralized interface formation. Direct evidence specifically demonstrating improvement in receiving-bone quality following chitosan-based biomaterial treatment remains limited and heterogeneous. Consequently, favorable inflammatory, molecular, or soft-tissue responses should not automatically be interpreted as evidence of successful tendon–bone integration when the biological quality of the receiving bone has not been adequately characterized [17,28,30,49,54].

Chitosan-containing composites may support tendon–bone healing through combinations of structural stabilization, localized bioactive factor delivery, cellular retention, matrix organization, and modulation of the regenerative environment [27,29,38,39,40,50,51,52]. However, effective biological integration requires coordinated interaction between regenerated soft tissue, fibrocartilage, mineralized interface, and receiving bone rather than the improvement of any single component. In this context, the osteochondral interface represents the critical transition through which tissue-specific matrix composition, mineralization, cellular organization, vascular support, and mechanical continuity are progressively integrated [2,3,4,11,17]. Therefore, evaluation of chitosan-based tendon–bone regeneration should incorporate assessment of receiving-bone quality and remodeling together with osteochondral interface maturation and structural integration, alongside molecular, histological, and biomechanical outcomes. This integrated assessment is necessary to determine whether biomaterial-induced improvements in the local regenerative environment ultimately translate into durable tendon–bone attachment and functional load transfer.

### 4.5. Biomechanical Restoration

Biomechanical restoration represents a critical functional outcome of tendon–ligament regeneration because biological repair becomes clinically meaningful when regenerated tissue can withstand physiological loading, transmit mechanical forces, and maintain structural stability [2,3,4,7,10]. Histological or molecular improvement alone therefore cannot establish complete regenerative success without corresponding assessment of functional parameters such as tensile strength, stiffness, load-to-failure, elasticity, and tendon–bone integration [10,29,40,51,55]. Biomechanical recovery reflects the cumulative contribution of immune regulation, vascularization, ECM remodeling, fibrocartilage maturation, enthesis regeneration, and tendon–bone healing rather than an isolated mechanical phenomenon [9,11,13,14,17]. Accordingly, biomechanical outcomes provide an important functional endpoint for determining whether molecular and structural improvements translate into mechanically competent regenerated tissue [12,13,14,15,18].

Evidence from chitosan–platelet composites, cell-containing hydrogels, bioadhesive matrices, and other chitosan-containing multifunctional platforms indicates that improved biomechanical outcomes can accompany coordinated improvements in matrix organization, interface reconstruction, vascular support, and immune regulation [17,18,29,40,55]. Because many of these systems contain multiple biologically active components, however, improved mechanical performance should be interpreted as an integrated platform effect rather than as evidence of an intrinsic biomechanical effect of chitosan alone [17,18,29,40]. These findings are consistent with the proposed Cascade Theory and ITLU framework, in which biomechanical restoration emerges from coordinated immune–vascular–matrix interactions, but they should not be regarded as direct experimental validation of these conceptual models [3,9,13,14,17]. A comparative qualitative synthesis of chitosan-based regenerative platforms across these interconnected domains is presented in Figure 5, illustrating the transition from predominantly structural systems toward increasingly multifunctional immunoregenerative strategies.

### 4.6. Integrated Interpretation

The evidence across ECM remodeling, fibrocartilage formation, enthesis regeneration, tendon–bone healing, and biomechanical restoration indicates that regenerative outcomes emerge from interconnected biological and functional processes rather than a single molecular pathway [1,9,10,11,12,13,14,17]. The comparative evidence is summarized in Table 3.

Taken together, these findings provide conceptual support for the proposed Immunoregenerative Cascade Theory and ITLU as complementary frameworks for organizing current evidence rather than as experimentally validated causal models [3,9,11,13,14]. The Cascade Theory provides a temporal interpretation of interconnected regenerative events, whereas the ITLU provides a spatial and functional framework for conceptualizing reciprocal interactions among immune, vascular, cellular, extracellular, and biomechanical domains [9,12,14,15,17]. From a translational perspective, this synthesis supports an evolution from predominantly scaffold-centered tissue engineering toward multifunctional and increasingly precision-oriented immunoregenerative strategies integrating biomaterial design with immune phenotyping, multi-omics technologies, computational approaches, and patient-specific regenerative engineering [3,4,9,12,13,14,15,17,18]. The integrated conceptual framework presented in Figure 6 summarizes this proposed evolution from biomaterial development through immune and mechanistic regulation toward systems-level integration and translational precision medicine while highlighting a roadmap for future validation rather than an established clinical pathway [3,9,14,17,18].

## 5. Translational Evidence from Chitosan-Based Composite Platforms

### 5.1. PRP/PRF Composite Systems

Chitosan-based composites incorporating platelet-rich plasma (PRP) or platelet-rich fibrin (PRF) represent clinically relevant regenerative platforms because they combine the material properties of chitosan with autologous platelet-derived bioactive mediators [27,29,51,52]. Preclinical evidence indicates that chitosan–platelet composites can support collagen organization, extracellular matrix maturation, tendon attachment, and interface reconstruction, while sustained retention of platelet-derived mediators may contribute to prolonged regenerative signaling within the local microenvironment [29,50,51,52]. Because these systems contain multiple biologically active components, however, their regenerative effects should be interpreted as composite-platform outcomes rather than attributed exclusively to the intrinsic immunomodulatory properties of chitosan [27,29,51,52]. Collectively, available evidence supports chitosan–platelet composites as a biologically integrated strategy combining structural support with localized regenerative signaling, although the relative contribution of individual material and platelet-derived components remains incompletely defined [9,11,15,29,33].

From a translational perspective, platelet-based chitosan composites may offer practical advantages through autologous preparation, favorable biocompatibility, and potential compatibility with existing surgical workflows [3,5,29,51,52]. Nevertheless, variability in platelet preparation, growth factor composition, chitosan formulation, and bioactive-factor incorporation [56,58], as well as differences in experimental models and outcome assessment [57,59,60], complicates direct comparison among studies and currently limits conclusions regarding comparative clinical superiority. Standardized preparation protocols, quantitative characterization of platelet products and chitosan formulations, longitudinal immune phenotyping, functional outcome assessment, and appropriately designed large-animal studies are therefore required before reproducible clinical translation can be established [13,14,17,59,60]. Accordingly, platelet-based chitosan composites should be regarded as promising and comparatively clinically adaptable platforms rather than as an established translational benchmark for all immunoregenerat ive biomaterials [9,12,14,15].

### 5.2. Stem Cell-Loaded Composite Systems

Stem cell-loaded chitosan composite systems integrate the structural and biological properties of chitosan-containing matrices with the differentiation capacity and paracrine activity of stem or progenitor cells [2,3,39,40,53]. Experimental evidence from tendon-derived stem cells and mesenchymal stem cells incorporated into chitosan-containing hydrogels indicates potential improvements in cellular retention, collagen organization, lineage-specific differentiation, interface regeneration, and biomechanical outcomes compared with corresponding acellular or control conditions [30,38,39,40,53]. These outcomes likely reflect combined contributions from biomaterial architecture, cellular activity, paracrine signaling, and modulation of the regenerative microenvironment rather than an intrinsic effect of chitosan alone [9,11,12,13,14,39,40]. Stem cell-loaded chitosan composites can therefore be interpreted as multifunctional cellular–biomaterial platforms capable of integrating structural support with biologically active tissue-regenerative mechanisms [3,9,11,13,15].

Despite encouraging preclinical findings, the translational maturity of stem cell-loaded systems remains limited by donor-dependent heterogeneity, manufacturing complexity, cell expansion requirements, quality control, regulatory considerations, long-term biosafety, and economic feasibility [2,3,8,13]. These limitations are particularly important when interpreting experimental efficacy because favorable outcomes obtained under controlled preclinical conditions do not necessarily establish scalability or clinical effectiveness [3,8,57,59,60]. Further standardization, mechanistic characterization, long-term functional assessment, and clinically relevant large-animal validation are therefore required before these platforms can progress toward routine clinical application [13,14,17,18,40]. Stem cell-loaded chitosan composites should consequently be regarded as biologically promising but translationally developing strategies rather than as platforms with established superiority over simpler regenerative approaches [3,9,12,14,15].

### 5.3. Hydrogel Platforms

Chitosan-based hydrogels provide hydrated three-dimensional matrices capable of supporting cellular retention, localized biomolecule delivery, nutrient diffusion, and modulation of the regenerative microenvironment [30,38,47,61]. Their physicochemical and mechanical properties can be modified through formulation, crosslinking, incorporation of bioactive components, and degradation characteristics, allowing for adaptation to different tissue-engineering requirements [22,32,47]. Preclinical studies of chitosan-containing hydrogel systems report improvements in selected outcomes, including extracellular matrix organization, cellular activity, interface healing, and biomechanical parameters, although these effects vary according to formulation and incorporated therapeutic components [30,38,39,40,47]. Accordingly, chitosan hydrogels are more appropriately regarded as adaptable delivery and regenerative matrices than as intrinsically immunoregenerative platforms independent of their composition [3,9,38,47].

Functionalized chitosan-containing hydrogels incorporating cells, platelet-derived mediators, or immunomodulatory components further illustrate the capacity of hydrogel engineering to integrate structural support with controlled biological signaling [14,39,40,43,47]. Evidence from broader non-chitosan hydrogel and controlled-delivery systems may provide contextual support for spatiotemporal therapeutic delivery, angiogenic modulation, and immune regulation but should not be interpreted as direct evidence of chitosan-specific biological effects [17,48,49]. From a translational perspective, hydrogels offer substantial engineering flexibility but remain subject to challenges involving formulation reproducibility, mechanical stability, degradation control, sterilization, manufacturing scalability, and long-term functional validation [22,32,47]. Chitosan-based hydrogels should therefore be viewed as versatile engineering platforms with potential for incorporation into increasingly multifunctional immunoregenerative strategies rather than as an established foundational platform for future precision therapy [3,13,14,15,47].

### 5.4. Multifunctional and Immunoregulatory Composite Systems

Multifunctional chitosan-containing composites integrate structural support with one or more biological functions, including immune modulation, controlled therapeutic delivery, cellular support, angiogenic signaling, and extracellular matrix regulation [3,5,9,15,22]. Rather than targeting individual stages of healing, these systems are designed to influence multiple interconnected regenerative processes, although the relative contribution of chitosan and incorporated bioactive components varies substantially among individual platforms [11,12,13,14,16]. Available preclinical evidence suggests that combining complementary structural and biological functions may broaden the range of regenerative domains addressed by a single platform, but this should not be interpreted as evidence that increasing material complexity necessarily produces superior therapeutic efficacy [3,9,14,15,17]. Multifunctionality is therefore best defined by coordinated biological functionality rather than simply by the number of incorporated components [3,13,14,15,18].

Recent chitosan-containing and broader immunoregulatory platforms illustrate strategies integrating immune modulation, controlled delivery, vascular regulation, matrix remodeling, and tissue-specific differentiation [14,17,18,43,49]. Non-chitosan systems within this literature provide important contextual evidence for broader design principles but should not be used to establish chitosan-specific therapeutic effects [14,49]. Increasing platform complexity may provide greater biological versatility while simultaneously introducing challenges in reproducibility, manufacturing, characterization, regulatory approval, scalability, and long-term safety [3,14,17,47,57]. The comparative characteristics of scaffold-based, platelet-containing, stem cell-loaded, hydrogel-based, and multifunctional platforms are therefore summarized in Table 4 as a qualitative translational comparison rather than a formal ranking of therapeutic superiority [9,13,14,15,17].

### 5.5. Critical Appraisal: Contradictory Findings and Sources of Heterogeneity

Despite growing evidence supporting chitosan-based composite biomaterials for tendon–ligament regeneration (Table 5 and Table 6), substantial heterogeneity remains in regenerative efficacy, mechanistic outcomes, and translational relevance [8,14,17,23,57]. Differences among reported outcomes may reflect variations in chitosan molecular weight, degree of deacetylation, crosslinking, degradation behavior, mechanical properties, scaffold architecture, and formulation parameters, which can influence material–cell interactions and regenerative performance [22,32,47]. Importantly, the available evidence does not demonstrate uniformly concordant improvements across regenerative domains. Willbold et al. reported favorable cellular infiltration, vascular ingrowth, and tendinous tissue formation with a chitosan-coated PCL scaffold, whereas biomechanical parameters did not significantly differ between implant groups and median force at failure remained lower than that of suture-only repair. Similarly, Çatal et al. [62] observed favorable histological and biochemical responses following chitosan application, but biomechanical differences did not reach statistical significance. Chevrier et al. further showed that chitosan–PRP improved selected MRI and histopathological outcomes, whereas chitosan formulated without PRP did not differ significantly from standard-of-care controls. Collectively, these neutral or discordant findings indicate that regenerative effects are outcome-, formulation-, and component-dependent and caution against attributing composite-system efficacy exclusively to chitosan [16,26].

Methodological heterogeneity in species, injury models, biomaterial fabrication, implantation procedures, follow-up duration, and outcome assessment further limits cross-study comparison and may contribute to apparently divergent findings [3,8,23,57,59,60]. The predominance of small-animal models and short observation periods also limits assessment of long-term mechanical adaptation and clinical durability. These limitations emphasize that molecular or histological improvement should not be interpreted as complete functional regeneration and support the need for standardized material characterization, integrated outcome assessment, and clinically relevant longitudinal validation [8,14,17,59,60].

### 5.6. Why Do Some Chitosan Platforms Fail? A Mechanistic Failure Analysis

Suboptimal outcomes may arise from interacting material, biological, temporal, and functional constraints. Material-related factors include variability in molecular characteristics, crosslinking, degradation kinetics, mechanical behavior, scaffold architecture, and release profiles [22,32,47]. Biological failure may result from persistent inflammatory activation, inadequate resolution, oxidative stress, insufficient vascularization, or dysregulated matrix remodeling [16,19,20,21,43,44]. A temporal mismatch between biomaterial degradation, bioactive factor release, and tissue remodeling may further disrupt regenerative progression even when individual biological responses are favorable [14,30,38,47]. Finally, failure may become apparent only at downstream functional levels when molecular or histological improvement does not translate into mature fibrocartilage, tendon–bone integration, or mechanical competence [10,14,17,29,40,55]. Thus, failure analysis should be used to identify specific material–biological mismatches rather than be interpreted as evidence of failure of a single immunoregenerative pathway [9,13,14,15].

### 5.7. Evidence Hierarchy of Chitosan-Based Composite Platforms

Figure 7 provides a structured qualitative hierarchy of chitosan-based platforms according to the breadth, consistency, directness, and translational maturity of available evidence across regenerative domains. Multifunctional, hydrogel-based, platelet-containing, cellular, and structural platforms show different evidence profiles, but greater platform complexity should not be interpreted as greater therapeutic efficacy or translational readiness [3,14,17,18,29,40,51]. The hierarchy therefore represents an evidence-mapping framework rather than a formal ranking, quantitative meta-analysis, or certainty-of-evidence assessment and is intended primarily to identify areas of relative evidence strength and persistent translational gaps [3,13,15,17,18].

### 5.8. Strength-of-Evidence Matrix

Table 7 summarizes the relative strength of evidence across individual biological and functional domains using structured qualitative categories based on consistency, mechanistic support, integration of molecular, histological, and functional outcomes, and translational relevance. The matrix indicates comparatively stronger support for macrophage regulation, inflammatory signaling, ECM remodeling, tendon–bone healing, and biomechanical outcomes, whereas fibrocartilage maturation, enthesis reconstruction, long-term interface adaptation, and clinically relevant ligament repair remain less extensively characterized [4,17,28,38,42]. These categories are intended to facilitate interpretation of the current evidence landscape rather than represent numerical scores, formal certainty-of-evidence grading, or definitive therapeutic ranking [3,8,14,15,57].

## 6. Current Challenges and Future Perspectives

### 6.1. What Is Still Missing?

Despite advances in chitosan-based biomaterials for tendon–ligament regeneration, important biological, material, and methodological gaps remain. From a biological perspective, the temporal and functional relationships linking immune regulation with vascular adaptation, cellular responses, ECM remodeling, interface maturation, and biomechanical recovery remain incompletely resolved, and many studies continue to evaluate these domains as separate endpoints [1,9,11,12,13]. From a material perspective, substantial heterogeneity persists in chitosan formulation and physicochemical characteristics, including molecular weight, degree of deacetylation, crosslinking, degradation behavior, and scaffold architecture, limiting direct comparison of biological responses across studies [14,19,20,21,22,32,47]. These differences also complicate interpretation of whether observed immunomodulatory effects arise from chitosan itself or from formulation-specific properties and incorporated components.

Methodological limitations further constrain interpretation and reproducibility. Preclinical studies vary in experimental models, injury characteristics, sampling time points, outcome definitions, and combinations of molecular, histological, and biomechanical assessments [8,12,13,14,15,17]. Consequently, favorable findings in individual molecular or histological endpoints cannot consistently be linked to subsequent tissue integration or durable functional recovery [3,10,14,17,18]. Addressing these gaps requires greater integration of biological, material, and methodological characterization so that the relationships among material properties, biological responses, and functional outcomes can be evaluated more consistently across chitosan-based regenerative platforms [8,14,57,59,60]. The principal biological, material, and methodological gaps identified across the current evidence base are summarized in Figure 8.

### 6.2. Emerging Omics Technologies for Precision Immunoregenerative Biomaterials

Emerging single-cell, spatial, proteomic, and metabolomic technologies may provide complementary approaches for resolving the biological complexity of tendon–ligament regeneration beyond conventional bulk molecular or histological assessment [3,9,13,14,15]. Single-cell transcriptomics can characterize cellular heterogeneity and identify distinct immune, stromal, and progenitor-cell populations; spatial transcriptomics can map cell-specific transcriptional states within the anatomical context of the regenerating tissue and clarify spatial relationships among immune, vascular, cellular, and ECM domains; proteomics can characterize changes in cytokines, signaling proteins, ECM components, and other extracellular or intracellular mediators; and metabolomics can provide information on metabolic states associated with inflammation, oxidative stress, cellular adaptation, and tissue remodeling [12,13,14,17,18]. Integration of these complementary datasets may therefore improve mechanistic resolution and identify stage-specific molecular and cellular signatures associated with regenerative responses. However, their application to chitosan-based tendon–ligament regeneration remains emerging, and evidence from broader regenerative biomaterial research should be considered contextual rather than direct validation of chitosan-specific mechanisms [12,13,14,17,18]. The principal applications and current conceptual roles of these emerging omics technologies in tendon–ligament regeneration are summarized in Table 8.

### 6.3. Translational Priorities and Future Directions

Future translation of chitosan-based tendon–ligament biomaterials will require standardized material characterization, longitudinal validation, clinically relevant large animal models, and robust biomechanical assessment [8,14,57,59,60]. Standardized reporting of molecular weight, degree of deacetylation, formulation, crosslinking, degradation behavior, architecture, mechanical properties, and bioactive components is necessary to improve reproducibility and permit meaningful comparison among platforms [14,19,20,21,22,32,47]. Longitudinal studies should integrate molecular, histological, ECM, enthesis, and functional outcomes across relevant stages of healing rather than relying on isolated endpoint measurements [8,12,13,14,15,17]. Validation in clinically relevant large animal models is additionally important to determine whether findings observed in small animal models remain applicable in tissues with more comparable anatomical, mechanical, and loading characteristics. Biomechanical assessment should be incorporated as a functional endpoint, including appropriate measures of stiffness, tensile properties, load-bearing capacity, and tendon–bone integration, because molecular or histological improvement alone does not establish durable functional regeneration [10,14,17,18]. Collectively, these priorities would strengthen reproducibility, establish relationships between biological and functional outcomes, and improve the translational maturity of chitosan-based immunoregenerative platforms.

### 6.4. Toward Precision Immunoregenerative Tendon–Ligament Engineering

Future chitosan-based biomaterials may ultimately move from empirically optimized multifunctional scaffolds toward mechanism-guided and adaptive biomaterial designs that are informed by the biological requirements of different stages of tissue healing [3,9,14,15,17,18]. Rather than simply increasing the number of incorporated bioactive components, mechanism-guided design would seek to align defined material properties, degradation behavior, bioactive factor presentation, and spatial or temporal delivery with specific regenerative processes, while adaptive platforms could be engineered to respond to changing features of the local microenvironment during inflammation, resolution, matrix remodeling, and interface maturation [1,9,11,13,14]. Such approaches could provide a rational framework for coordinating immune regulation with ECM remodeling, vascular adaptation, cellular activity, and biomechanical restoration. However, adaptive or precision biomaterials remain a future design paradigm rather than an established therapeutic capability, and their proposed responsiveness, reproducibility, safety, and functional benefit require prospective mechanistic and translational validation before clinical application [12,14,15,17,18]. Recent studies further illustrate this emerging design trajectory, including biomimetic chitosan-based fibers for tendon regeneration [56], chitosan-assisted tendon healing after primary repair [62], and chitosan–platelet-rich plasma systems for tendon repair [64], alongside related immunomodulatory and functionalized fibrous biomaterial platforms [65,66]. The conceptual transition toward mechanism-guided and adaptive immunoregenerative engineering is illustrated in Figure 9.

## 7. Conclusions

Tendon–ligament regeneration is increasingly understood as an integrated process involving immune regulation, vascular adaptation, cellular responses, extracellular matrix remodeling, interface maturation, and biomechanical recovery. Current preclinical evidence indicates that chitosan-based composite biomaterials can influence several of these regenerative domains, particularly when structural and biological functions are combined. However, the heterogeneity of chitosan formulations, physicochemical properties, experimental models, and outcome measures limits direct comparison across studies and prevents definitive conclusions regarding the comparative efficacy or chitosan-specific contribution of multicomponent platforms.

The Immunoregenerative Cascade Theory and Immunoregenerative Tendon–Ligament Unit (ITLU) provide complementary, hypothesis-generating frameworks for organizing the temporal and spatial–functional interactions underlying tendon–ligament regeneration. These frameworks should not be interpreted as validated causal or predictive models. Future research should prioritize standardized characterization of chitosan-based materials, longitudinal integration of molecular, histological, ECM, interface, and biomechanical outcomes, clinically relevant large-animal validation, and emerging multi-omics approaches to improve mechanistic resolution. Such efforts may facilitate the development of mechanism-guided and increasingly adaptive chitosan-based biomaterials while establishing whether immunoregulatory effects can be translated into durable structural and functional tendon–ligament regeneration.

## Figures and Tables

**Figure 1:**
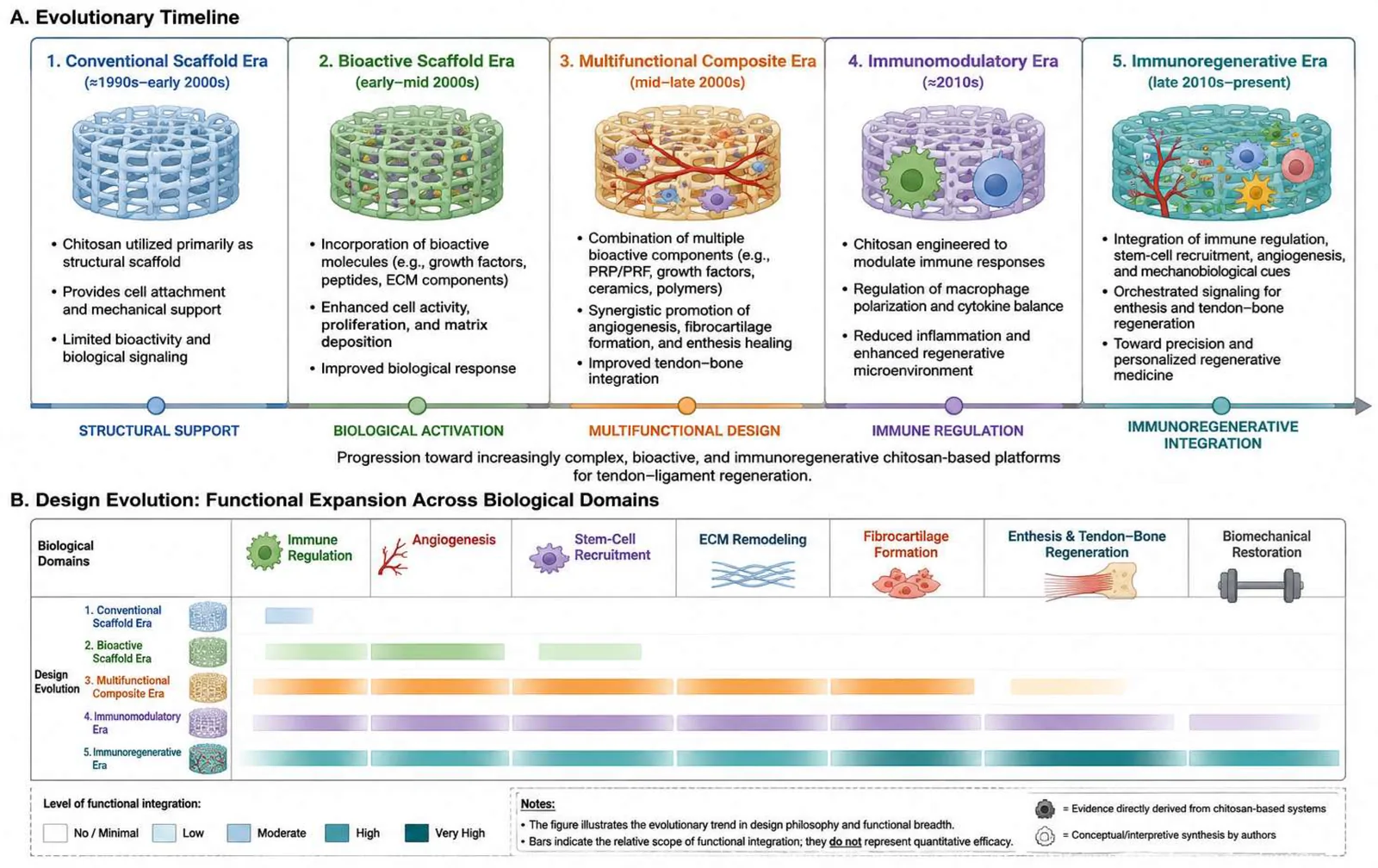
Evolution of chitosan-based composite biomaterials for tendon–ligament regeneration: (**A**) evolutionary timeline; (**B**) design evolution: functional expansion across biological domains.

**Figure 2:**
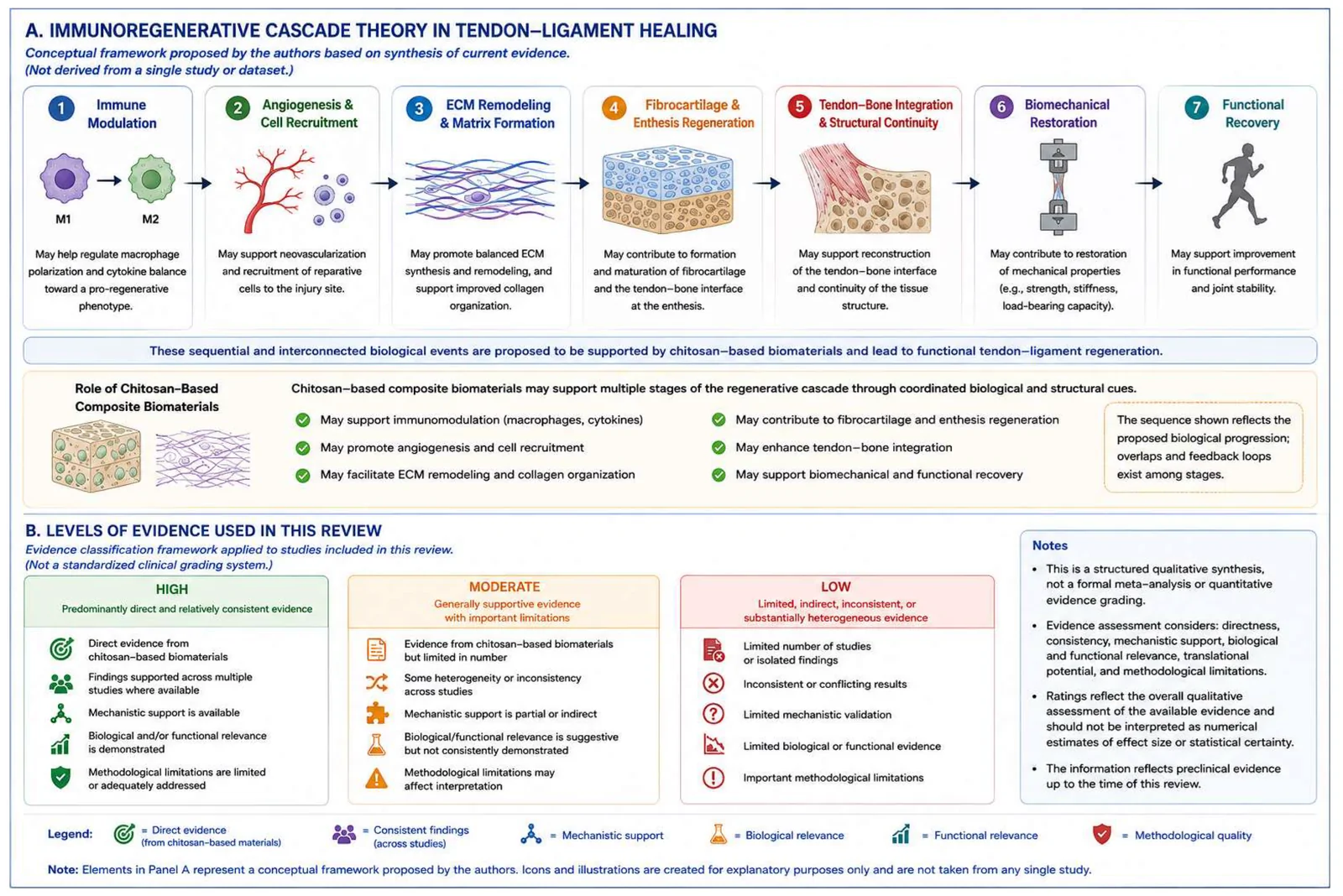
Immunoregenerative Cascade Theory of tendon–ligament healing: from immune reprogramming to biomechanical restoration. (**A**) Conceptual framework of the proposed Immunoregenerative Cascade Theory, illustrating the sequential and interconnected biological stages of tendon–ligament healing and the proposed roles of chitosan-based composite biomaterials in supporting immune modulation, angiogenesis and cell recruitment, ECM remodeling, fibrocartilage and enthesis regeneration, tendon–bone integration, biomechanical restoration, and functional recovery. (**B**) Evidence-classification framework used in this review to characterize the strength and nature of the available evidence across the included studies; this framework is a qualitative synthesis developed for this review and is not a standardized clinical grading system.

**Figure 3:**
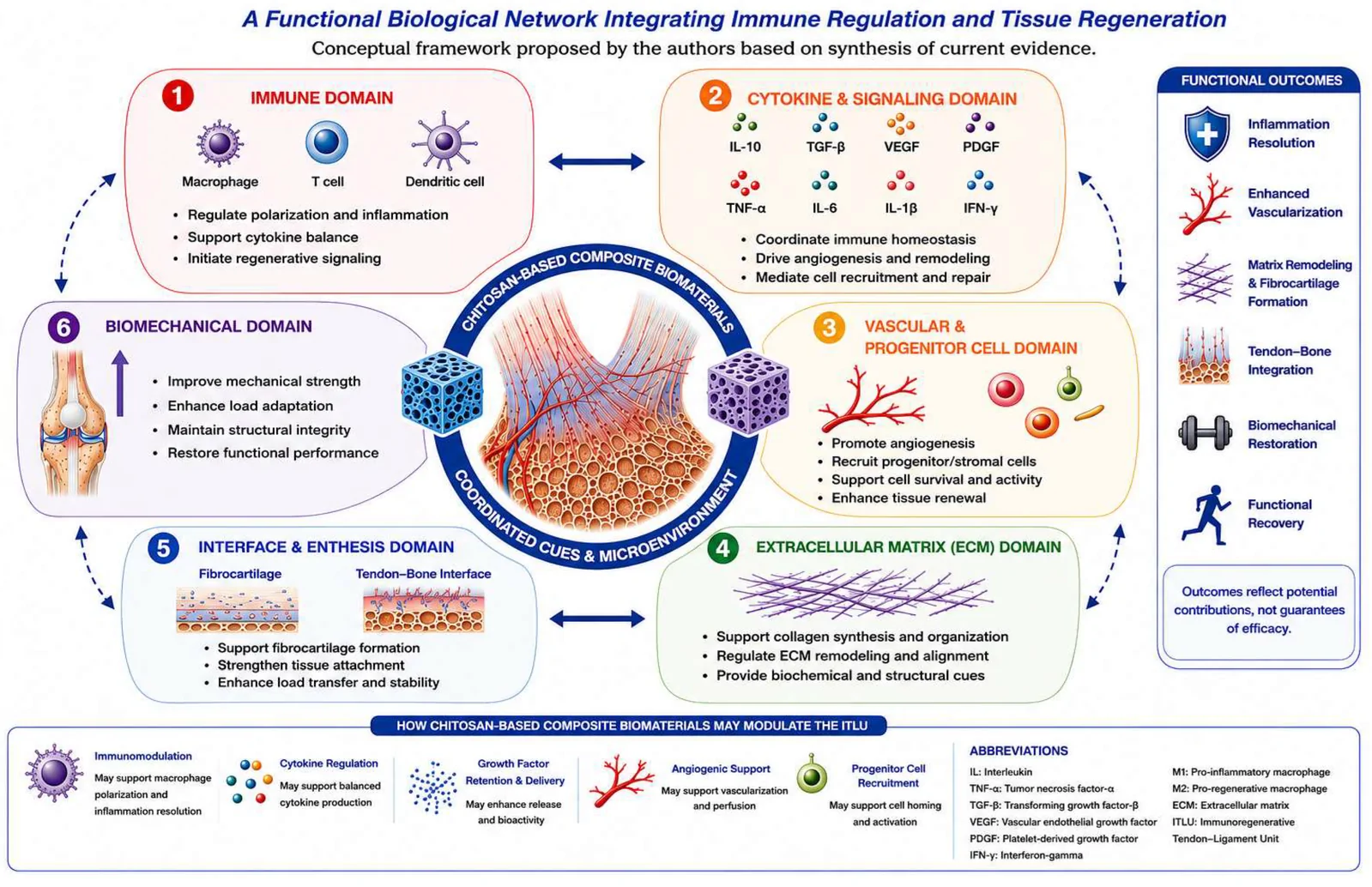
Immunoregenerative Tendon–Ligament Unit (ITLU): a conceptual framework integrating immune regulation and tissue regeneration. The framework represents a conceptual synthesis proposed by the authors based on available evidence and should not be interpreted as a validated causal pathway or quantitative model. Solid arrows indicate proposed sequential or directional relationships between interconnected regenerative domains, whereas dashed arrows indicate proposed modulatory or cross-domain interactions between these domains. These arrow relationships are conceptual representations of the proposed framework and should not be interpreted as experimentally validated causal relationships.

**Figure 4:**
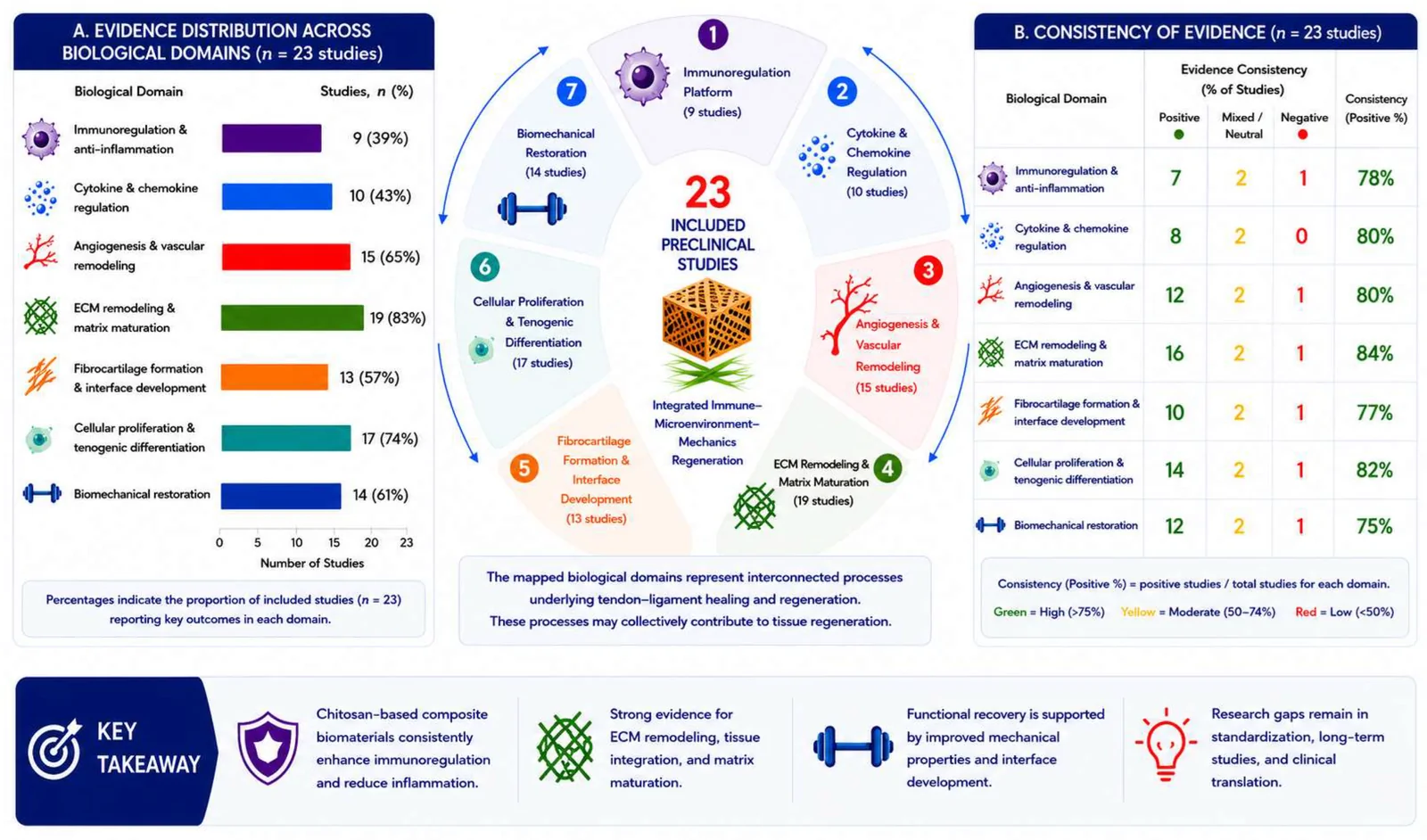
Evidence mapping of chitosan-based composite biomaterials for tendon–ligament regeneration. Qualitative synthesis of published preclinical evidence across biological domains, animal models, biomaterial platforms, and research gaps. The mapping reflects evidence derived from the cited literature, whereas its organization and integrated interpretation represent the authors’ conceptual synthesis and should not be interpreted as a quantitative evidence-grading system.

**Figure 5:**
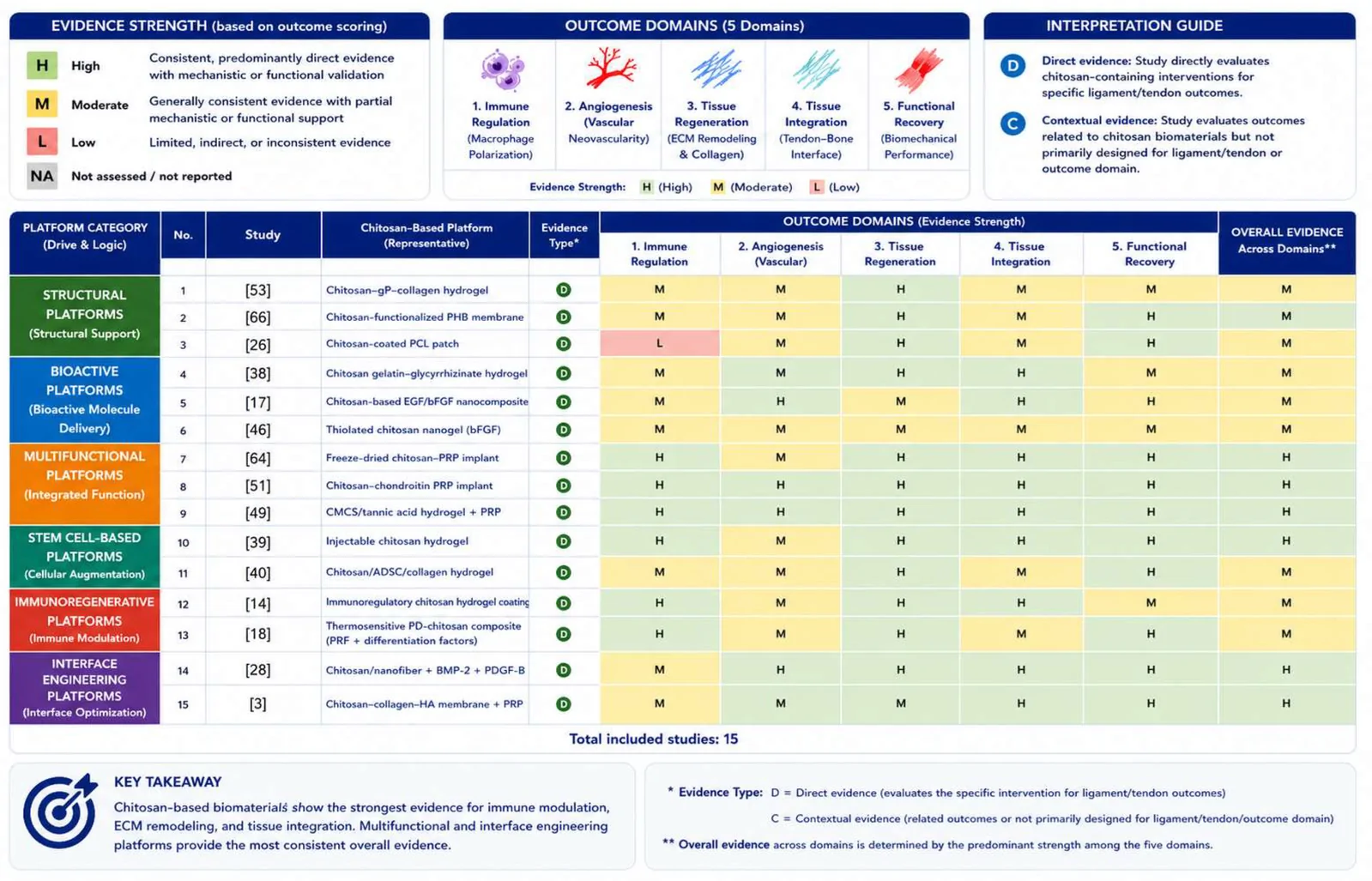
Qualitative evidence mapping of chitosan-based biomaterial platforms for tendon–ligament regeneration. Comparative synthesis of published preclinical evidence illustrating the relative support for major chitosan-based biomaterial platforms across interconnected regenerative domains, including immune modulation, angiogenesis, extracellular matrix remodeling, fibrocartilage formation, tendon–bone integration, and biomechanical restoration. The comparative patterns reflect the consistency and convergence of findings reported across the cited studies rather than quantitatively pooled effect estimates. The organization and cross-domain interpretation of the evidence represent the authors’ conceptual synthesis and are intended to illustrate the progressive transition from predominantly structural biomaterials toward multifunctional immunoregenerative platforms. Accordingly, the figure should be interpreted as a qualitative comparative framework rather than a formal ranking or quantitative evidence-grading system.

**Figure 6:**
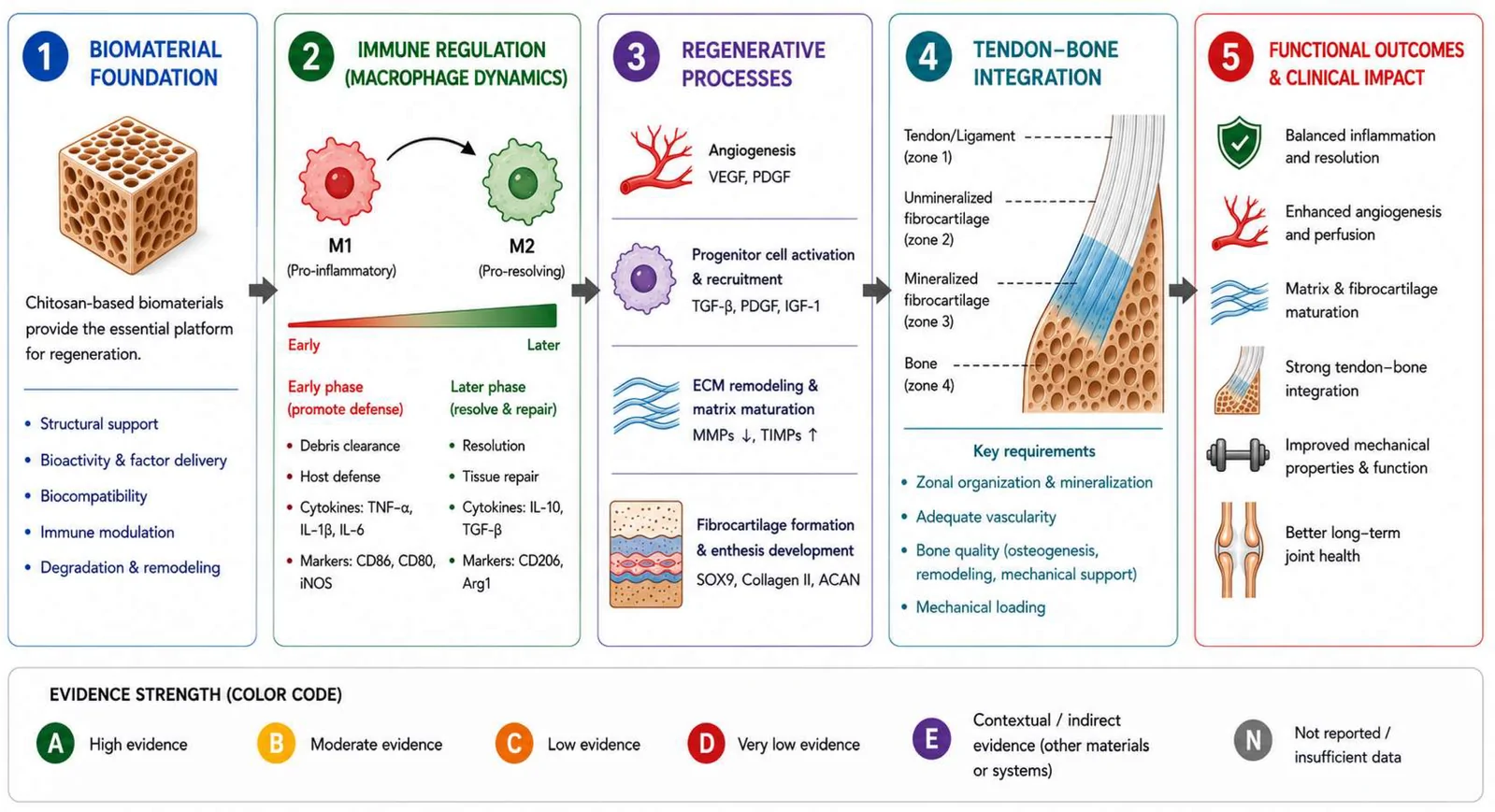
Evidence-informed roadmap for chitosan-based immunoregenerative biomaterials in tendon–ligament regeneration. The arrows indicate the proposed progression and interrelation-ships among biomaterial design, immune regulation, regenerative processes, tendon–bone integra-tion, and functional outcomes. Solid arrows indicate proposed directional or sequential relation-ships between the major stages, whereas dashed lines indicate proposed modulatory or cross-domain interactions. These relationships represent an evidence-informed conceptual framework and should not be interpreted as experimentally validated causal pathways or quanti-tative relationships.

**Figure 7:**
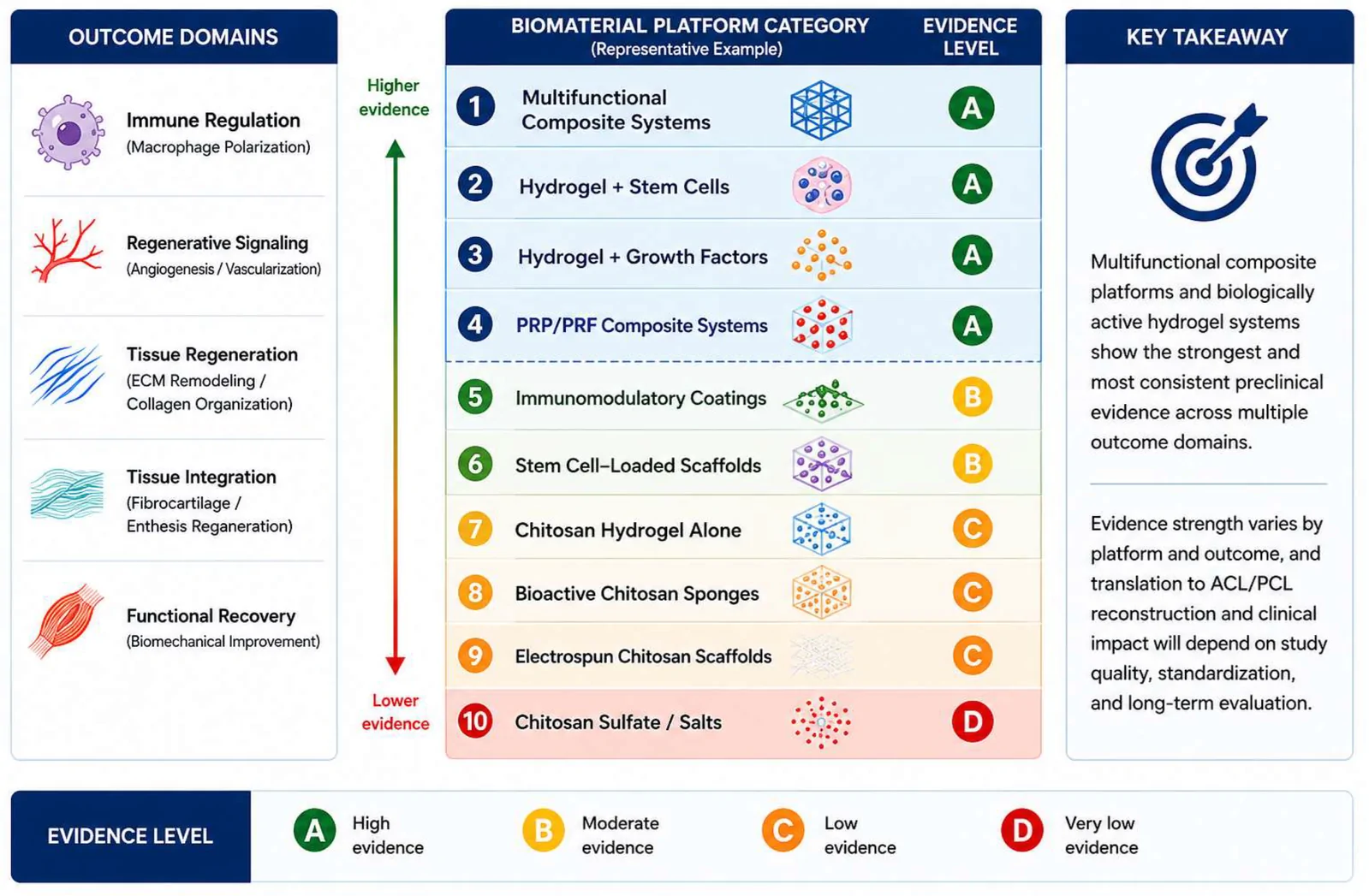
Structured qualitative evidence hierarchy of chitosan-based biomaterial platforms for tendon–ligament regeneration. This figure integrates representative published preclinical evidence across five interconnected regenerative domains: immune regulation, regenerative signaling, tissue regeneration, tissue integration, and functional recovery. Biomaterial platforms are comparatively positioned according to the consistency and directness of reported findings, mechanistic support, cross-domain convergence of molecular, histological, and biomechanical outcomes, and translational relevance. Evidence levels (A–D) represent structured qualitative descriptors derived from the literature synthesis, whereas the hierarchical organization and cross-platform interpretation represent the authors’ conceptual synthesis. Accordingly, the hierarchy is intended to visualize relative evidence maturity and identify current evidence gaps rather than to establish biomaterial superiority, quantitative effect estimates, or formal certainty-of-evidence grading.

**Figure 8:**
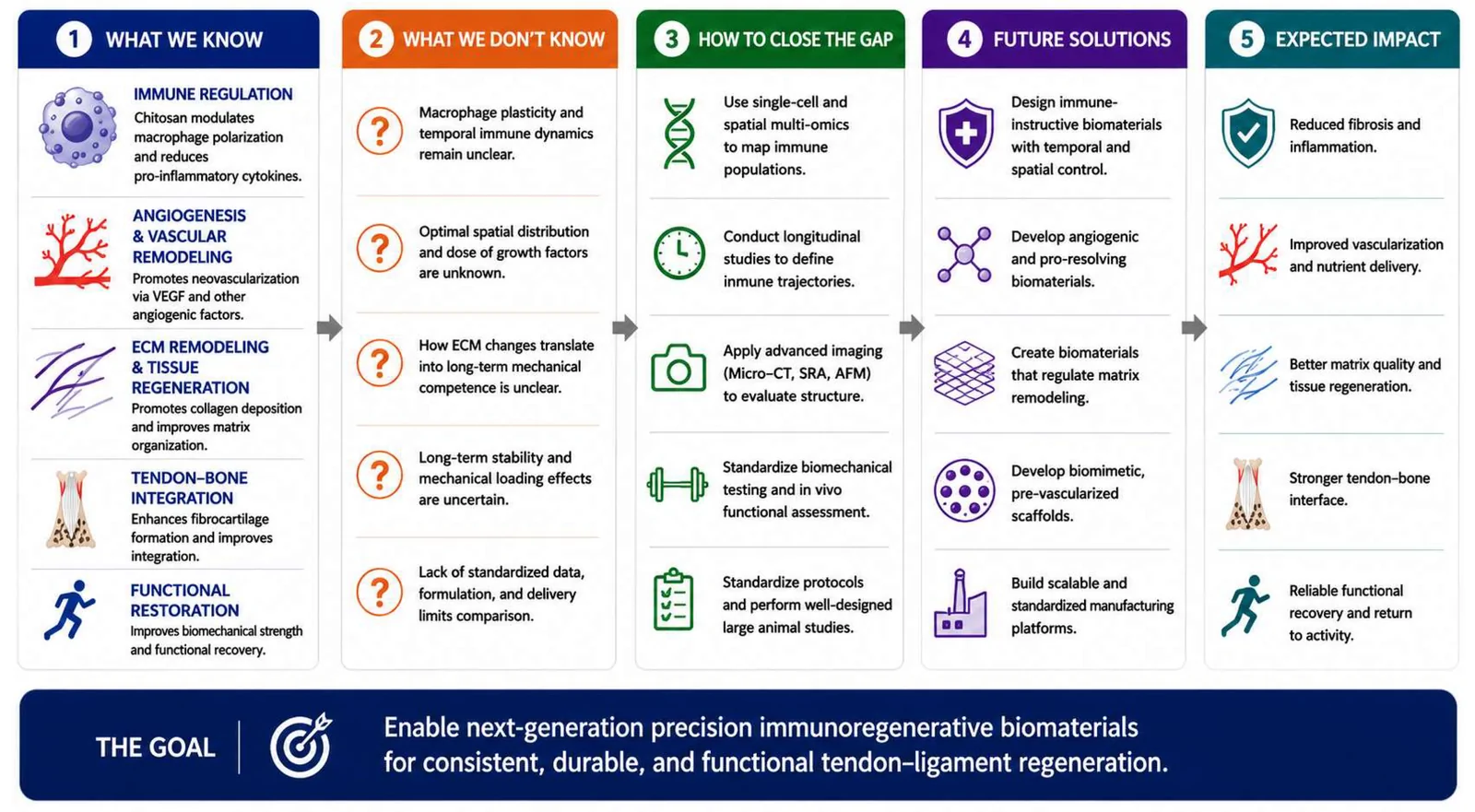
Knowledge gaps and future research priorities in chitosan-based composite biomaterials for tendon–ligament regeneration.

**Figure 9:**
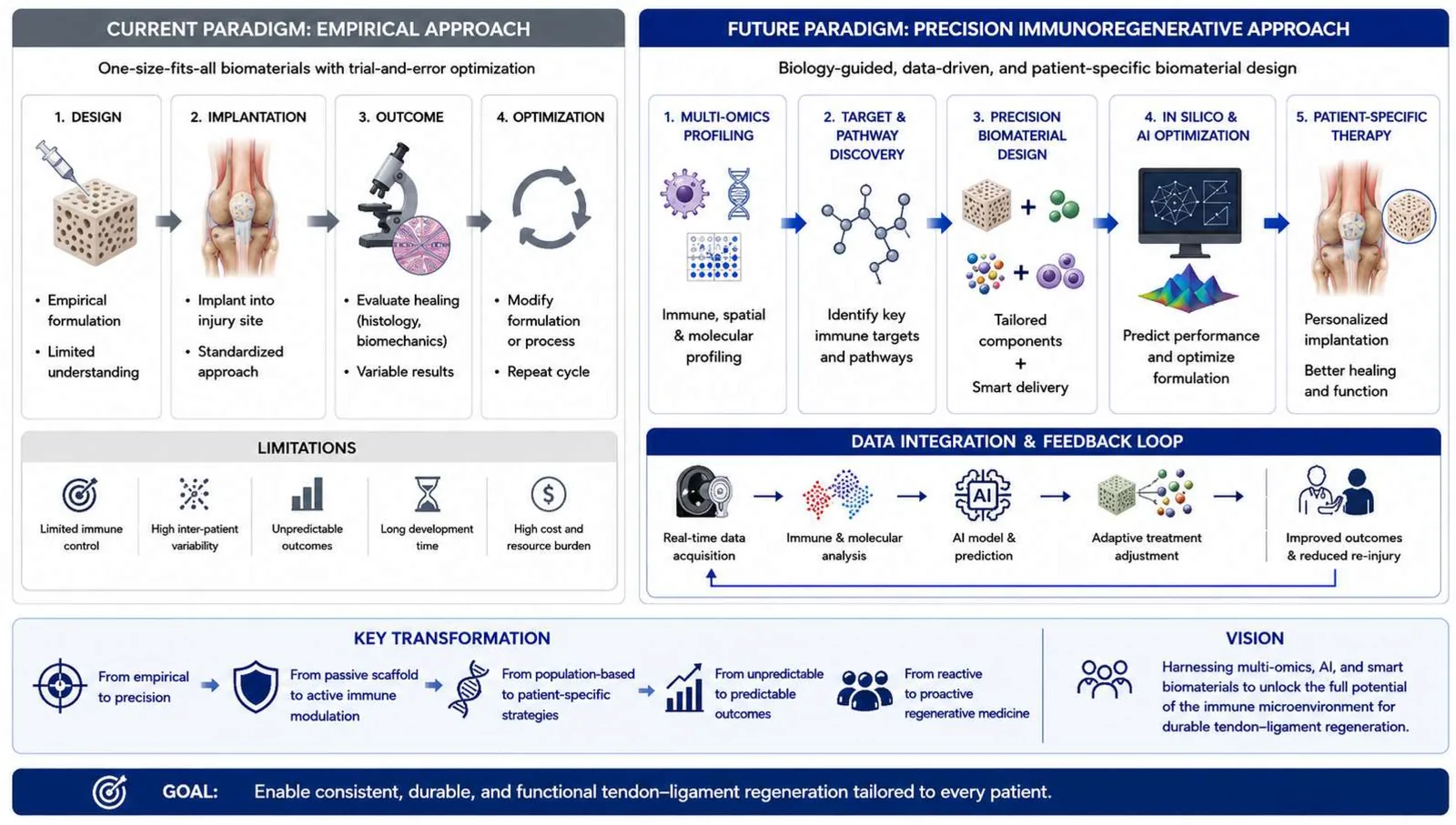
From empirical biomaterials to precision immunoregenerative medicine. Solid dark-blue arrows indicate the proposed process flow and indicate feedback loops between the monitoring, molecular analysis, model refinement, and adaptive treatment stages. The arrows represent conceptual relationships within the proposed roadmap and should not be inter-preted as validated causal pathways or clinical workflows.

**Table 1:** Direct Evidence for Chitosan-Based Biomaterial Platforms and Contextual Comparison with Alternative Biomaterials Relevant to Tendon–Ligament Regeneration.

Evidence Category	Study/Reference	Biomaterial Platform	Experimental Context	Principal Characteristics/Function	Principal Outcomes or Advantages	Main Limitations/Interpretation	Evidence Category
Direct chitosan evidence	[3]	Chitosan–collagen–hydroxyapatite nanofibers + PRP	Rabbit; rotator cuff repair	Nanofibrous composite combining chitosan, collagen, hydroxyapatite, and PRP	Histological improvement, collagen organization, and osteotendinous regeneration	Multiple active components complicate attribution	Direct chitosan evidence
Direct chitosan evidence	[14]	Chitosan-containing immunoregulatory hydrogel coating	Rabbit; ACL reconstruction	Surface hydrogel designed to modulate local immune response and graft–bone interaction	Macrophage regulation, scar reduction, and graft–bone integration	Immunomodulatory effects remain formulation- and context-dependent	Direct chitosan evidence
Direct chitosan evidence	[17]	Chitosan-stabilized BSA–VEGF nanoparticles in graded porous scaffold	Rabbit; tendon–bone interface	Chitosan-mediated nanoparticle delivery within graded scaffold	Angiogenesis, collagen I deposition, Sharpey-like fibers, and osseointegration	Complex delivery system; multiple components contribute to outcome	Direct chitosan evidence
Direct chitosan evidence	[18]	Thermosensitive hydroxybutyl-chitosan core–shell microneedle	Rat; Achilles tendon injury	Implantable chitosan-based controlled-delivery platform	Inflammation control, collagen alignment, tenogenic differentiation, and biomechanics	Novel platform requiring further reproducibility and translational validation	Direct chitosan evidence
Direct chitosan evidence	[26]	Chitosan-coated PCL graft	Rat; rotator cuff repair	Electrospun PCL graft modified with chitosan coating	Cellular infiltration and graft integration; favorable tissue response	Biomechanical outcomes were not uniformly superior to controls	Direct chitosan evidence
Direct chitosan evidence	[28]	HA/chitosan multilayer nanofilm on aligned PCL fibers	Rabbit; degenerative rotator cuff	Aligned fibrous scaffold with HA/chitosan surface nanofilm and VEGF	Angiogenesis, tenogenesis, collagen organization, and stiffness	Outcomes depend on scaffold architecture and incorporated VEGF	Direct chitosan evidence
Direct chitosan evidence	[29]	Chitosan–PRP implant	Sheep; rotator cuff repair	Autologous PRP incorporated into chitosan-based implant	Histological repair, tendon attachment, and mechanical restoration	Long-term translational reproducibility remains uncertain	Direct chitosan evidence
Direct chitosan evidence	[30]	Sulfhydrylated chitosan hydrogel + kartogenin/FGF-2	Rabbit; rotator cuff repair	Chemically modified chitosan hydrogel with controlled bioactive delivery	Tendon–bone regeneration, collagen deposition, and tissue integration	Multicomponent system; contribution of thiolation and incorporated factors is interdependent	Direct chitosan evidence
Direct chitosan evidence	[38]	Chitosan–gelatin–glycerophosphate hydrogel	Rabbit; tendon–bone healing	Injectable hydrogel providing structural support and localized biological activity	Tendon–bone continuity, collagen deposition, and interface healing	Primarily preclinical evidence; formulation-dependent response	Direct chitosan evidence
Direct chitosan evidence	[39]	Injectable chitosan hydrogel + tendon-derived stem cells	Rabbit; rotator cuff tendon–bone healing	Cell-compatible injectable chitosan platform	Histology, collagen maturation, tendon–bone healing, and biomechanical improvement	Cell–material interaction contributes substantially to outcome	Direct chitosan evidence
Direct chitosan evidence	[40]	Chitosan/β-GP/collagen hydrogel + MSCs	Rat; chronic Achilles tendon injury	Injectable cell-supportive chitosan hydrogel	Akt/GSK-3β signaling, ECM remodeling, and tendon regeneration	Cell-mediated effects limit attribution to chitosan alone	Direct chitosan evidence
Direct chitosan evidence	[46]	Thiolated-chitosan microgel composite coating	Rat; tendon–skin repair	Thiolated chitosan-based smart-suture/coating platform with bFGF and conductive component	Collagen organization, tenogenic response, and tissue repair	Multifactorial intervention; electrical/conductive contribution must be considered	Direct chitosan evidence
Direct chitosan evidence	[49]	Carboxymethyl-chitosan/tannic-acid hydrogel + PRP	Rat; rotator cuff repair	Injectable multifunctional chitosan hydrogel	Tendon–bone interface regeneration, histology, and biomechanical outcomes	Multifunctional formulation; requires component-specific validation	Direct chitosan evidence
Direct chitosan evidence	[50]	Freeze-dried chitosan–PRP implant	Rabbit; rotator cuff repair	Implantable chitosan/PRP delivery platform	Tendon attachment, bone remodeling, and histological improvement	Follow-up and formulation characteristics vary across studies	Direct chitosan evidence
Direct chitosan evidence	[51]	Freeze-dried chitosan–thrombin–PRP implant	Rabbit; rotator cuff repair	Platelet-based chitosan implant with thrombin-mediated formulation	Tendon regeneration, collagen organization, histology, and biomechanics	Platelet and thrombin contributions complicate mechanistic attribution	Direct chitosan evidence
Direct chitosan evidence	[53]	Chitosan/β-glycerophosphate/collagen hydrogel	Rat; Achilles tendon defect	Injectable chitosan-based hydrogel incorporating collagen and tendon stem cells	Histological improvement, collagen organization, and biomechanical recovery	Multifactorial system; effects cannot be attributed exclusively to chitosan	Direct chitosan evidence
Direct chitosan evidence	[55]	Piezoelectric chitosan gel	Rat; Achilles tendon rupture	Injectable/local chitosan-based piezoelectric material	Inflammatory modulation, tendon-related gene expression, and biomechanical improvement	Experimental platform; translational maturity remains limited	Direct chitosan evidence
Direct chitosan evidence	[56]	Chitosan/γ-polyglutamic-acid interfacial fibers	Rabbit; tendon regeneration	Interfacial polyelectrolyte fiber system with silk-fibroin coating	Fiber alignment, biomimicry, collagen organization, and mechanical properties	Recent preclinical evidence; clinical translation remains unestablished	Direct chitosan evidence
Direct chitosan evidence	[62]	Chitosan	Rat; quadriceps tendon rupture	Local chitosan application following tendon repair	Histological response, inflammatory markers, collagen organization, and biomechanics	Effects were not uniformly demonstrated across all outcome domains	Direct chitosan evidence
Direct chitosan evidence	[63]	Chitosan/HA multilayer-coated PCL membrane	Rabbit; ACL reconstruction	Surface-engineered PCL membrane combining chitosan, HA, SDF-1α, and BMP-2	Fibrocartilage formation, tendon–bone integration, and biomechanical improvement	Multicomponent platform; chitosan-specific contribution is difficult to isolate	Direct chitosan evidence
Direct chitosan evidence	[64]	Freeze-dried chitosan–PRP implant	Sheep; rotator cuff repair	Platelet-based chitosan implant enabling localized delivery of autologous PRP-derived factors	Tendon attachment, collagen maturation, and histological repair	Pilot ovine evidence; preparation and platelet composition may vary	Direct chitosan evidence
Direct chitosan evidence	[65]	Collagen-enriched chitosan–PVA hydrogel + collagen-I/LR12	Rat; rotator cuff tendon injury	Injectable immunomodulatory hydrogel targeting TREM1-associated responses	Immunocompatibility, ECM/tendon regeneration, and modulation of inflammatory signaling	Recent preclinical evidence; requires independent validation and functional translation	Direct chitosan evidence
Direct chitosan evidence	[66]	Chitosan-functionalized electrospun PHB membrane/sleeve	Rabbit; ACL reconstruction	Implantable chitosan-functionalized fibrous membrane/sleeve with PRP-derived growth-factor contribution	Tendon–bone integration, fixation strength, and fibrocartilage formation	Composite effects and contribution of surface functionalization require separation	Direct chitosan evidence
Contextual alternative platform	Collagen [3,40,53]	Collagen-based or collagen-containing matrices	Tendon/ligament regeneration	Native ECM structural protein with strong cell-adhesive properties	Biological compatibility, cellular attachment, and matrix organization	Standalone collagen may have limited mechanical stability and relatively rapid degradation	Contextual alternative platform
Contextual alternative platform	GelMA	Gelatin methacryloyl hydrogel	Tendon/ligament tissue engineering	Photocrosslinkable gelatin-derived hydrogel	Tunable mechanical properties, cell encapsulation, and biofabrication compatibility	Properties depend strongly on degree of functionalization and crosslinking	Contextual alternative platform
Contextual alternative platform	PLGA	Poly(lactic-co-glycolic acid)	Regenerative biomaterials	Biodegradable synthetic copolymer	Controllable degradation and established delivery capability	Acidic degradation products and limited intrinsic biological signaling	Contextual alternative platform
Contextual alternative platform	PCL [26,28,63]	Polycaprolactone-based scaffolds	Tendon/ligament repair	Slowly degradable synthetic polyester with good processability	Mechanical durability and suitability for electrospun/aligned scaffolds	Slow degradation and limited intrinsic bioactivity often necessitate functionalization	Contextual alternative platform
Contextual alternative platform	Hyaluronic acid (HA) [28,63]	HA-based or HA-containing systems	Tendon–bone regeneration	Native ECM glycosaminoglycan with high hydration capacity	Hydrated matrix environment and compatibility with surface functionalization	Relatively weak mechanical properties and rapid degradation when used alone	Contextual alternative platform
Contextual alternative platform	Silk fibroin [56]	Silk-fibroin-containing fibrous systems	Tendon regeneration	Protein biomaterial with strong fibrous architecture	Favorable mechanical properties, biocompatibility, and scaffold processability	Biological performance depends on processing, structure, and functionalization	Contextual alternative platform
Contextual alternative platform	Decellularized ECM (dECM)	Tissue-derived decellularized matrix	Tendon/ligament regeneration	Native tissue-derived matrix retaining biochemical and structural cues	Tissue-specific biochemical signals and native-like architecture	Batch variability, residual immunogenicity, sterilization, and standardization challenges	Contextual alternative platform

Note: Direct chitosan evidence refers to studies in which chitosan is an explicit component of the investigated biomaterial platform. Contextual alternative-platform evidence refers to biomaterials that provide relevant structural, biological, or engineering characteristics but are not interpreted as direct evidence of chitosan-specific effects. The table is intended as a qualitative contextual synthesis rather than a systematic head-to-head comparison or quantitative ranking of biomaterial superiority.

**Table 2:** Core immunoregenerative design principles for next-generation tendon–ligament biomaterials.

Design Principle	Biological Target	Key Mechanism	Desired Regenerative Outcome	Representative Studies
Macrophage Reprogramming	M1 → M2 transition	Resolution of inflammation and restoration of macrophage homeostasis	Reduced fibrosis and enhanced regenerative microenvironment	[14,18,49,62,65]
Cytokine Balancing	TNF-α ↓, IL-1β ↓, IL-10 ↑, TGF-β ↑	Restoration of inflammatory homeostasis and pro-regenerative signaling	Controlled healing progression and reduced chronic inflammation	[14,18,38,49]
Inflammatory Signaling Control	NF-κB ↓, NLRP3 ↓, TLR4-related signaling	Suppression of persistent inflammatory signaling and inflammasome activation	Protection from matrix degradation and promotion of regenerative healing	[14,18,55,65]
Oxidative Stress Regulation	Nrf2-related antioxidant response	Reduction of oxidative stress and enhancement of cellular survival	Improved cellular viability and ECM synthesis	[18,55,56]
Angiogenic Remodeling	VEGF ↑, vascular maturation	Promotion of vascularization and nutrient delivery during tissue remodeling	Enhanced tissue maturation and vascular support	[3,17,28,51,66]
Stem-Cell Recruitment and Retention	MSC/TDSC recruitment, survival, and paracrine signaling	Chitosan-mediated cell retention and regenerative niche formation	Enhanced cellular replenishment and regenerative capacity	[39,40,53]
ECM Guidance	COL I ↑, COL III ↓; collagen organization	Regulation of collagen deposition, alignment, and matrix maturation	Functional collagen architecture and improved tissue quality	[26,28,55,56,66]
Fibrocartilage Formation	SOX9, aggrecan, fibrocartilage maturation	Promotion of interface-specific matrix differentiation	Regeneration of fibrocartilage and improved load transfer	[3,29,38,49,50]
Enthesis Reconstruction	Mineralized fibrocartilage and tendon–bone interface	Coordinated matrix maturation and interface regeneration	Restoration of tendon–bone continuity and biological fixation	[3,29,38,50,51]
Mechanical Adaptation	Load-responsive remodeling	Integration of matrix maturation with mechanical loading	Biomechanical restoration and functional tissue adaptation	[17,18,26,51,55]
Controlled Bioactive Delivery	Sustained/spatiotemporal release of regenerative mediators	Localized retention and controlled presentation of growth factors/platelet-derived signals	Prolonged regenerative signaling and improved tissue regeneration	[17,30,38,46,49,51]
Precision Personalization	Immune/microenvironment-responsive biomaterial design	Integration of multifunctional biomaterials with adaptive biological regulation	More predictable and patient-tailored regenerative responses	[14,18,46,65]

Note: ↑ denotes increased or upregulated expression/activity, whereas ↓ denotes decreased or downregulated expression/activity, relative to the relevant baseline or control condition. The arrow (→) in “M1 → M2 transition” denotes a phenotypic shift from a predominantly pro-inflammatory M1 macrophage state toward a reparative M2-associated phenotype.

**Table 3:** Comparative qualitative evidence assessment of chitosan-based biomaterial platforms for tendon–ligament regeneration.

Platform Category	Representative Studies *	Principal Regenerative Strategy	Consistent Biological Outcomes	Consistent Functional Outcomes	Evidence Strength
Chitosan-based structural and fibrous scaffolds	[26,28,56,63,66]	Structural support, biomimetic fiber organization, cell guidance, and bioactive surface functionalization	Improved cell attachment, collagen deposition, tenogenic organization, and scaffold–tissue integration	Improved structural organization and tendon/tendon–bone integration; biomechanical effects remain variable	Moderate
Chitosan hydrogel platforms	[30,38,46,55,65]	Injectable/localized delivery, microenvironment regulation, controlled bioactive delivery, and matrix support	Enhanced ECM remodeling, cellular activity, collagen organization, vascularization, and regenerative signaling	Improved tissue organization and biomechanical recovery where evaluated	Moderate
Chitosan + stem/progenitor cell systems	[39,40,53]	Combination of chitosan-mediated microenvironmental support with regenerative cell delivery	Enhanced tenogenic differentiation, collagen organization, matrix maturation, and tissue regeneration	Improved structural healing and load-bearing capacity	High
Chitosan–platelet concentrate (PRP/PRF) composites	[3,29,49,50,51,52,64]	Sustained retention and delivery of platelet-derived and regenerative mediators within chitosan-containing matrices	Increased collagen deposition/maturation, angiogenesis, fibrocartilage formation, and regenerative tissue organization	Improved tendon attachment, tendon–bone integration, structural repair, and biomechanical recovery	High
Chitosan-containing growth-factor delivery systems	[17,46,49,54]	Controlled/localized delivery of bFGF, VEGF, platelet-derived factors, and other regenerative mediators	Enhanced angiogenesis, tenogenic/osteogenic responses, ECM remodeling, and interface maturation	Improved tendon–bone/interface regeneration and functional tissue restoration	Moderate
Immunoregulatory and bioactive chitosan platforms	[14,18,49,65]	Modulation of the inflammatory microenvironment coupled with pro-regenerative signaling	Improved inflammatory regulation, ECM remodeling, collagen organization, and regenerative microenvironment formation	Improved healing quality and structural restoration	Moderate
Multifunctional chitosan-containing composite platforms	[17,18,46,51,54,66]	Integration of structural support, controlled bioactive delivery, immune/microenvironment regulation, angiogenesis, and regenerative signaling	Coordinated enhancement of ECM remodeling, cellular recruitment, vascularization, collagen organization, and tissue maturation	Improved structural integration and overall regenerative performance	Moderate

* Representative studies selected from the included evidence to illustrate each biomaterial platform; not an exhaustive list of all studies in the category.

**Table 4:** Comparative critical analysis of chitosan-based regenerative platforms for tendon–ligament healing.

No.	Included Study	Chitosan-Based Platform	Principal Regenerative Function	Main Strengths	Key Limitations/Critical Considerations	Translational Relevance
1.	[3]	Chitosan–collagen–hydroxyapatite nanofibers + PRP	Structural regeneration and tendon–bone integration	Biomimetic ECM components combined with platelet-derived factors	Multicomponent system makes mechanism attribution difficult	Moderate–High
2.	[14]	Chitosan-containing immunoregulatory hydrogel coating	Macrophage regulation and scar reduction	Directly addresses immune microenvironment and graft–bone integration	ACL reconstruction model; immunological endpoints require standardized cross-study comparison	High
3.	[17]	Chitosan-stabilized BSA–VEGF nanoparticles in graded porous scaffold	Angiogenesis and tendon–bone interface regeneration	Spatially organized scaffold with controlled VEGF presentation and vascular/osseointegration outcomes	Highly complex platform; relevance to partial ligament healing remains indirect	Moderate
4.	[18]	Thermosensitive hydroxybutyl-chitosan core–shell microneedle + PRP	Sequential inflammatory control and tissue regeneration	Integrates temporal delivery, immune regulation, and tenogenic remodeling	Complex manufacturing and implantation strategy; limited long-term translation	Moderate
5.	[26]	Chitosan-coated PCL graft	Structural scaffold integration	Combines mechanically stable PCL with chitosan surface bioactivity	Chitosan is primarily a coating; long-term biological contribution remains uncertain	Moderate
6.	[28]	HA/chitosan multilayer nanofilm on aligned PCL fibers	Angiogenesis and tenogenesis	Combines aligned topography with bioactive nanofilm; supports collagen organization and vascularization	Degenerative rotator-cuff model may not directly represent partial ligament injury	Moderate
7.	[29]	Chitosan–PRP implant	Platelet-mediated regeneration and tendon attachment	Large-animal sheep model; clinically relevant repair setting and structural outcomes	PRP preparation and animal-specific biology introduce variability	High
8.	[30]	Sulfhydrylated chitosan hydrogel + kartogenin/FGF-2	Controlled bioactive delivery and tendon–bone regeneration	Injectable multifunctional hydrogel with sustained regenerative signaling	Multiple active components complicate identification of the chitosan-specific effect	Moderate–High
9.	[38]	Chitosan–gelatin–glycerophosphate hydrogel + collagenase	Local matrix remodeling and tendon–bone healing	Injectable thermosensitive platform with localized enzymatic activity	Collagenase-mediated mechanism may not translate directly to ligament regeneration	Moderate
10.	[39]	Injectable chitosan hydrogel + tendon-derived stem cells	Cellular augmentation and tendon–bone regeneration	Integrates chitosan matrix with tendon-derived progenitor cells and structural outcomes	Cell source and delivery conditions may limit reproducibility	Moderate–High
11.	[40]	Chitosan/β-GP/collagen hydrogel + MSCs	Cell-mediated ECM remodeling and tenogenesis	Provides mechanistic evidence involving Akt/GSK-3β signaling	Chronic Achilles model; pathway findings require validation in other injury models	Moderate–High
12.	[46]	Thiolated-chitosan microgel smart-suture system + bFGF/electrical stimulation	Localized bioactive delivery and tendon repair	Combines chitosan adhesivity, growth-factor delivery, conductive stimulation, and structural repair	Multifunctionality increases manufacturing and regulatory complexity	Moderate
13.	[49]	Carboxymethyl-chitosan/tannic-acid hydrogel + PRP	Immunomodulation, osteogenic response, and tendon–bone regeneration	Integrates chitosan derivative, antioxidant/functional chemistry, and PRP; evaluates structural and biomechanical endpoints	Complex composite; causal contribution of individual components remains difficult to establish	Moderate–High
14.	[50]	Freeze-dried chitosan–PRP implant	Tendon attachment and bone remodeling	Rabbit transosseous repair model; direct structural and histological evaluation	Short-to-intermediate follow-up and repair-model specificity	High
15.	[51]	Freeze-dried chitosan–thrombin–PRP implant	Sustained platelet-mediated regeneration	Combines chitosan retention with thrombin/PRP-mediated regenerative signaling; rabbit rotator-cuff model	Multifactorial composition and translational standardization of platelet products remain challenges	High
16.	[53]	Chitosan/β-glycerophosphate/collagen hydrogel + tendon stem cells	Cell-supportive matrix and tenogenic regeneration	Combines injectable chitosan matrix with stem-cell augmentation; improved collagen organization and biomechanical healing	Rat Achilles model; composition and cell effects are difficult to separate	Moderate
17.	[55]	Piezoelectric chitosan gel	Regenerative signaling, antibacterial activity, and mechanical stimulation	Multifunctional platform integrating tissue repair with electromechanical functionality	Technological complexity and limited long-term translational evidence	Moderate
18.	[56]	Chitosan/γ-polyglutamic-acid interfacial fibers	Biomimetic ECM organization and mechanical support	Fiber-based biomimicry and interfacial polyelectrolyte design	Very recent evidence; long-term in-vivo and translational validation remains limited	Moderate
19.	[62]	Chitosan	Local modulation of tissue healing	Direct evaluation of chitosan in tendon repair with histological, inflammatory, and biomechanical outcomes	Relatively simple platform; mechanism of immunomodulation requires deeper molecular validation	Moderate
20.	[63]	Chitosan/HA multilayer-coated PCL membrane	Tendon–bone interface and fibrocartilage regeneration	Biomimetic multilayer architecture with controlled presentation of bioactive factors	Complex coating architecture and reconstruction-specific model	Moderate
21.	[64]	Freeze-dried chitosan–PRP implant	Sustained platelet-derived regenerative signaling	Large-animal ovine model; clinically relevant rotator-cuff repair context	PRP composition and freeze-drying may introduce biological variability	High
22.	[65]	Collagen-enriched chitosan–PVA hydrogel + LR12	Immunomodulation and ECM regeneration	Directly investigates inflammatory signaling together with ECM/tendon regeneration	Early preclinical evidence; pathway and long-term functional validation remain necessary	Moderate
23.	[66]	Chitosan-functionalized electrospun PHB membrane/sleeve + PRP-derived factors	Tendon–bone interface regeneration and fixation	Combines structural fibrous support with bioactive growth-factor presentation	Multifactorial platform limits attribution of benefit specifically to chitosan	Moderate

**Table 5:** Translational gap analysis of chitosan-based composite biomaterials for tendon–ligament regeneration.

Biological Target/Innovation	Current Preclinical Evidence	Major Translational Gap	Clinical Consequence	Priority Future Direction
Macrophage Polarization (M1→M2)	Strong evidence in small-animal models demonstrating reduced inflammation and improved tissue regeneration	Limited characterization of macrophage heterogeneity and temporal immune dynamics in large-animal and human tissues	Uncertain reproducibility of immunomodulatory effects in clinical settings	Single-cell and spatial immune profiling; large-animal validation
Cytokine Regulation	Consistent modulation of inflammatory and regenerative cytokines reported across multiple studies	Lack of standardized cytokine panels and longitudinal monitoring	Difficult comparison between studies and limited biomarker translation	Standardized immunological outcome measures
NF-κB and NLRP3 Modulation	Demonstrated suppression of chronic inflammatory signaling	Limited mechanistic validation linking pathway regulation to long-term functional outcomes	Unclear causal relationship between molecular changes and clinical benefit	Mechanistic studies integrating molecular and biomechanical endpoints
Angiogenic Remodeling	Improved vascularization frequently reported in composite platforms	Insufficient understanding of vascular maturation and long-term functionality	Enhanced vessel formation may not always translate into tissue regeneration	Longitudinal vascular imaging and functional perfusion assessment
Stem-Cell Recruitment and Retention	Strong evidence for enhanced cellular recruitment and regenerative activity	Limited knowledge regarding cell fate, persistence, and host-cell interactions	Unpredictable long-term regenerative contribution	Cell-tracking technologies and lineage mapping
ECM Remodeling	Consistent increases in collagen type I deposition and matrix organization	Lack of standardized ECM maturity assessment across studies	Difficult comparison of regenerative quality	Quantitative ECM maturation and collagen organization metrics
Fibrocartilage Formation	Frequently reported in advanced composite systems	Absence of uniform histological scoring systems	Variable interpretation of fibrocartilage regeneration	Consensus histological assessment criteria
Enthesis Regeneration	Promising restoration of tendon–bone transitional architecture	Limited evidence in clinically relevant large-animal models	Uncertain applicability to human reconstruction procedures	Large-animal translational studies
Tendon–Bone Healing	Strong histological and imaging evidence of interface improvement	Heterogeneous outcome measures and follow-up periods	Difficulty identifying optimal therapeutic platform	Standardized translational study design
Biomechanical Restoration	Improved tensile strength and load-to-failure reported in several studies	Histological improvements do not always correlate with mechanical recovery	Uncertain functional significance of biological findings	Integration of biomechanics with molecular and histological analyses
PRP/PRF Composite Systems	High translational feasibility due to clinical availability	Variability in platelet concentration and inconsistent biological responses between studies	Potential variability in clinical reproducibility	Standardization of preparation and characterization

**Table 6:** Evidence strength and translational maturity across biological domains of chitosan-based biomaterials for tendon–ligament regeneration.

Regenerative Domain	Directness to Chitosan	Evidence Consistency	Mechanistic Support	Functional Relevance	Translational Maturity	Principal Evidence Gap
Macrophage polarization and immune regulation	Direct	Consistent across multiple experimental platforms	Strong; supported by macrophage phenotyping and inflammatory-pathway analyses	High	Moderate	Limited longitudinal characterization of macrophage states and insufficient validation of spatial and temporal immune regulation
Inflammatory cytokine regulation	Direct	Consistent, although heterogeneous across formulations and models	Moderate–Strong; evidence includes cytokine and inflammatory-signaling outcomes	Moderate–High	Moderate	Limited standardization of cytokine panels, sampling time points, and biomaterial formulations
Oxidative-stress and inflammasome regulation	Direct/Contextual	Moderately consistent	Moderate; mechanistic evidence is available but remains limited across tendon–ligament models	Moderate	Low–Moderate	Limited direct validation of Nrf2/NLRP3-related mechanisms in clinically relevant ligament models
Angiogenic remodeling	Direct/Contextual	Generally consistent	Moderate; supported by angiogenic and growth-factor-related findings	Moderate–High	Moderate	Limited integration of vascular remodeling with immune and matrix outcomes over time
Extracellular matrix remodeling	Direct	Consistent across multiple platforms	Strong; supported by collagen deposition, organization, and matrix-related outcomes	High	Moderate	Limited standardized assessment of matrix quality, fiber organization, and long-term tissue maturation
Fibrocartilage formation	Direct/Contextual	Moderately consistent	Moderate	High for interface regeneration	Low–Moderate	Relatively few studies provide standardized histological and molecular confirmation of mature fibrocartilage
Enthesis/tendon–bone interface regeneration	Direct/Contextual	Moderately consistent	Moderate	High	Low–Moderate	Limited long-term characterization of graded interface maturation and mineralized fibrocartilage restoration
Biomechanical restoration	Direct	Generally consistent when evaluated	Moderate	High	Moderate	Limited long-term biomechanical validation, standardized testing protocols, and correlation with molecular and histological outcomes
Immune–ECM crosstalk	Direct/Contextual	Emerging	Moderate	High	Low	Most studies assess immune and ECM outcomes separately rather than demonstrating bidirectional mechanistic coupling
Multi-omics and systems-level validation	Emerging/Contextual	Limited	Limited–Moderate	Potentially high	Low	Few studies integrate transcriptomic, proteomic, spatial, metabolic, and biomechanical datasets to guide biomaterial design

**Table 7:** Domain-based evidence assessment matrix for chitosan-based biomaterials in tendon–ligament regeneration.

Evidence Domain	Representative Studies	Consistency of Findings	Mechanistic Support	Translational Relevance	Strength of Evidence
Macrophage Polarization	[14,39,56,62,65]	High	High	High	High
NF-κB/NLRP3 Suppression	[14,39,56,65]	Moderate–High	Moderate–High	Moderate	Moderate
Cytokine Reprogramming	[14,26,40,50,56,65]	High	Moderate–High	High	High
Angiogenic Remodeling	[28,30,49,55,63,65]	High	High	High	High
Stem Cell Recruitment/Retention	[14,29,53]	Moderate	High	Moderate	Moderate
ECM Remodeling	[3,17,18,26,29,30,38,39,40,46,49,50,51,53,55,56,62,63,64,65,66]	High	High	High	High
Fibrocartilage Formation	[3,28,29,30,49,50,62,63,64,65]	Moderate	High	High	Moderate
Enthesis Regeneration	[3,28,29,30,49,50,62,63,65]	Moderate	Moderate–High	High	Moderate
Tendon–Bone Healing	[3,28,29,30,49,50,62,63,64,65]	High	High	High	High
Biomechanical Restoration	[3,17,18,26,28,29,30,38,39,40,46,49,50,51,55,56,62,64,65,66]	High	High	High	High
ACL/PCL Translational Potential	[13,63,65]	Moderate	Moderate	High	Moderate
Precision Immunoregenerative Medicine	[14,17,56,62,65]	Moderate	Moderate	High	Moderate

Note: Evidence categories represent a structured qualitative synthesis rather than formal quantitative grading. Moderate, Moderate–High, and High reflect increasing consistency across studies, mechanistic support, integration of molecular, histological, and functional outcomes, and translational relevance. These categories are comparative descriptors and should not be interpreted as numerical scores or formal levels of certainty.

**Table 8:** Emerging omics technologies and their applications in tendon–ligament regeneration.

Technology	Primary Information	Application in Regeneration	Potential Contribution to Biomaterial Design
Single-cell RNA Sequencing	Cell-specific gene expression	Identification of macrophage subsets and regenerative cells	Targeted immunomodulation
Spatial Transcriptomics	Spatial gene-expression mapping	Tendon–bone interface characterization	Spatially controlled biomaterials
Proteomics	Protein and cytokine networks	Functional pathway identification	Bioactive molecule selection
Metabolomics	Cellular metabolic states	Inflammation and regeneration profiling	Metabolic microenvironment regulation
Epigenomics	Chromatin and gene regulation	Cell-fate determination	Long-term regenerative programming
Multi-omics Integration	Systems-level biological networks	Comprehensive regenerative mapping	Precision biomaterial engineering

## Data Availability

No new data were created or analyzed in this study. Data sharing is not applicable to this article.

## Abbreviations

The following abbreviations are used in this manuscript:

ACL	Anterior Cruciate Ligament
Akt	Protein Kinase B
bFGF	Basic Fibroblast Growth Factor
COL I	Collagen Type I
COL III	Collagen Type III
ECM	Extracellular Matrix
IL	Interleukin
ITLU	Immunoregenerative Tendon–Ligament Unit
MMP	Matrix Metalloproteinase
MSC	Mesenchymal Stem Cell
NF-κB	Nuclear Factor Kappa B
NLRP3	NOD-, LRR-, and Pyrin Domain-Containing Protein 3
Nrf2	Nuclear Factor Erythroid 2-Related Factor 2
PCL	Posterior Cruciate Ligament
PDGF	Platelet-Derived Growth Factor
PI3K	Phosphoinositide 3-Kinase
PRF	Platelet-Rich Fibrin
PRP	Platelet-Rich Plasma
SOX9	SRY-Box Transcription Factor 9
TDSC	Tendon-Derived Stem Cell
TGF-β	Transforming Growth Factor Beta
TIMP	Tissue Inhibitor of Metalloproteinase
TLR4	Toll-Like Receptor 4
TNF-α	Tumor Necrosis Factor Alpha
VEGF	Vascular Endothelial Growth Factor
